# Economic evaluation of the TriageHF Plus clinical pathway for device-based remote monitoring in heart failure

**DOI:** 10.1136/heartjnl-2025-326702

**Published:** 2025-12-17

**Authors:** Fozia Z Ahmed, Rachel Harwood, David Lanctin, Catherine Leonard, Sarah Medland, Daniela Afonso, Stuart Mealing, Toni Weldon, Callum Blunt, Niall G Campbell, Joanne K Taylor

**Affiliations:** 1 Department of Cardiology, Manchester University NHS Foundation Trust, Manchester, UK; 2 Keele Cardiovascular Research Group, Keele University, Newcastle-under-Lyme, UK; 3 Statistics Department, Research and Innovation, Central Manchester University Hospitals NHS Foundation Trust, Manchester, UK; 4 Centre for Biostatistics, University of Manchester, Manchester, UK; 5 Medtronic, Mounds View, Minnesota, USA; 6 Enterprise House, York Health Economics Consortium Ltd, Heslington, UK; 7 Department of Cardiology, Northern Care Alliance NHS Foundation Trust, Manchester, UK; 8 Division of Cardiovascular Sciences, University of Manchester, Manchester, UK; 9 Division of Informatics, Imaging and Data Sciences, University of Manchester, Manchester, UK

**Keywords:** Health Care Economics and Organizations, Heart Failure, Telemedicine, Defibrillators, Implantable

## Abstract

**Background:**

In 2024, the UK National Institute of Health and Care Excellence (NICE) recommended TriageHF alerts as an option for remote monitoring of patients with heart failure (HF) and a compatible cardiac implantable electronic device (CIED). Data on the cost-effectiveness of this approach has not been published. This research evaluates the cost-effectiveness of TriageHF Plus in public hospital settings, using data from the TriageHF Plus multicentre study (758 participants, NCT04177199).

**Methods:**

An economic model was developed to capture the lifetime cost and benefits of TriageHF Plus versus usual care, based on a site of 300 eligible patients. Analysis on individual patient-level data informed model efficacy and resource use parameters. EuroQol five-dimensional questionnaire data and unit costs were obtained from published, peer-reviewed literature and national databases respectively. Costs and benefits were discounted at 3.5% per annum to adjust future costs and benefits to present value.

**Results:**

In a site size of 300, TriageHF Plus was predicted to prevent 384 (363–405) hospitalisations over 5 years. In total, TriageHF Plus saved £3989 (£1812–£5563) per person-lifetime versus usual care and was more effective and cost-saving in 99.4% of simulations. Results were robust to changes in key input parameters. Modelling showed that to avoid one hospitalisation, 1.7 people would need lifetime access to TriageHF Plus.

**Conclusion:**

The TriageHF Plus pathway is cost-effective for the remote monitoring of HF in patients with CIEDs.

**Trial registration number:**

NCT04177199.

**WHAT IS ALREADY KNOWN ON THIS TOPIC**

- The TriageHF Plus remote monitoring clinical pathway is effective at reducing unplanned hospitalisations in patients with heart failure (HF) and a cardiac implantable electronic device. The cost-effectiveness for use in the UK National Health Service has not yet been published.

**WHAT THIS STUDY ADDS**

- TriageHF Plus is not only cost-effective (as defined by the National Institute of Health and Care Excellence), but cost-saving by approximately £4000 per person-lifetime compared with care without device-based remote monitoring.

**HOW THIS STUDY MIGHT AFFECT RESEARCH, PRACTICE OR POLICY**

- This analysis demonstrates how adoption of TriageHF Plus can save the NHS money while maintaining safe, effective patient care.

## Introduction

 Heart failure (HF) is the leading cause of unplanned hospitalisations in the UK, costing approximately £400 million per year.^1^ Many HF hospitalisations are potentially avoidable if deterioration is identified early.^2–5^

**Figure 1:**
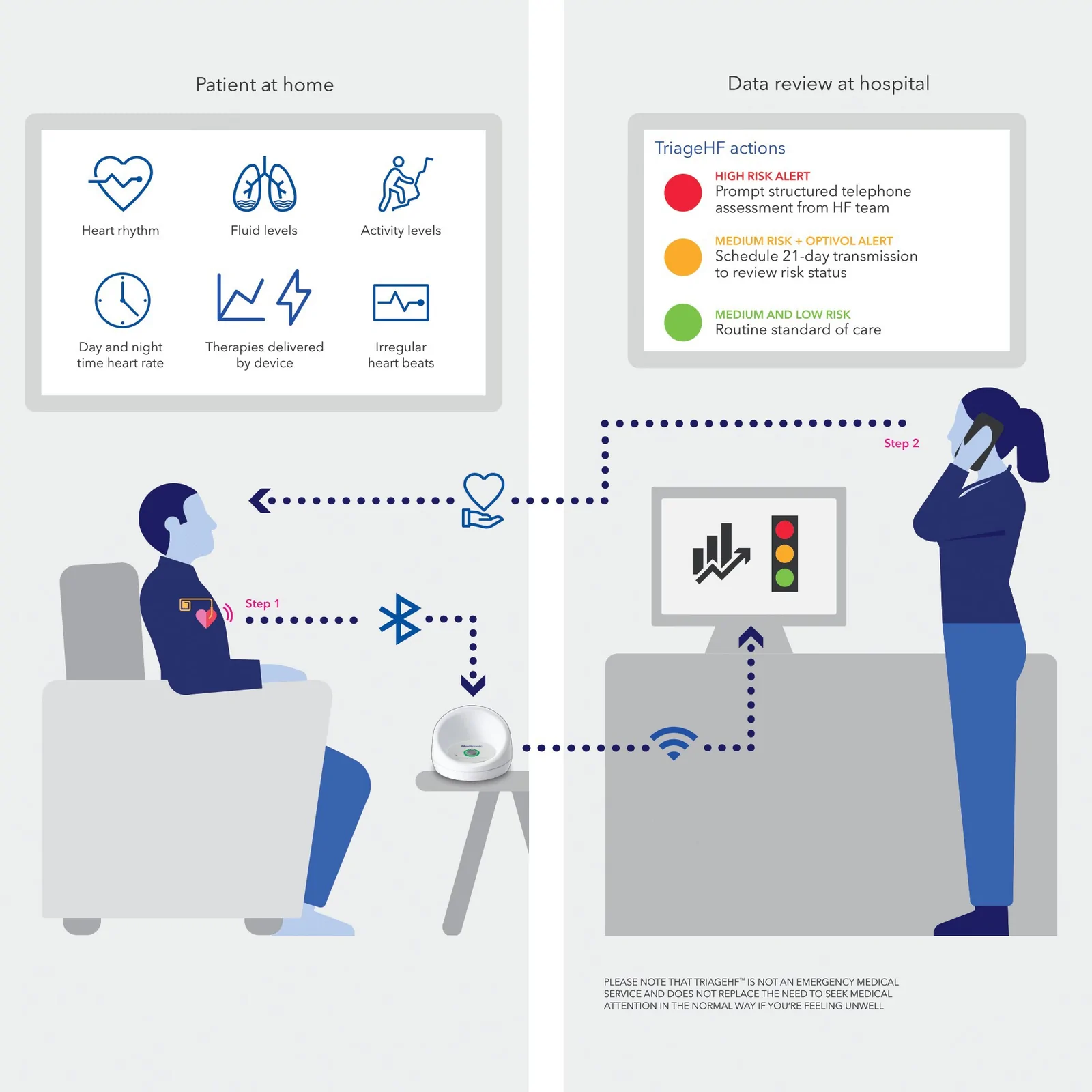
Schematic of the TriageHF Plus pathway.

**Figure 2:**
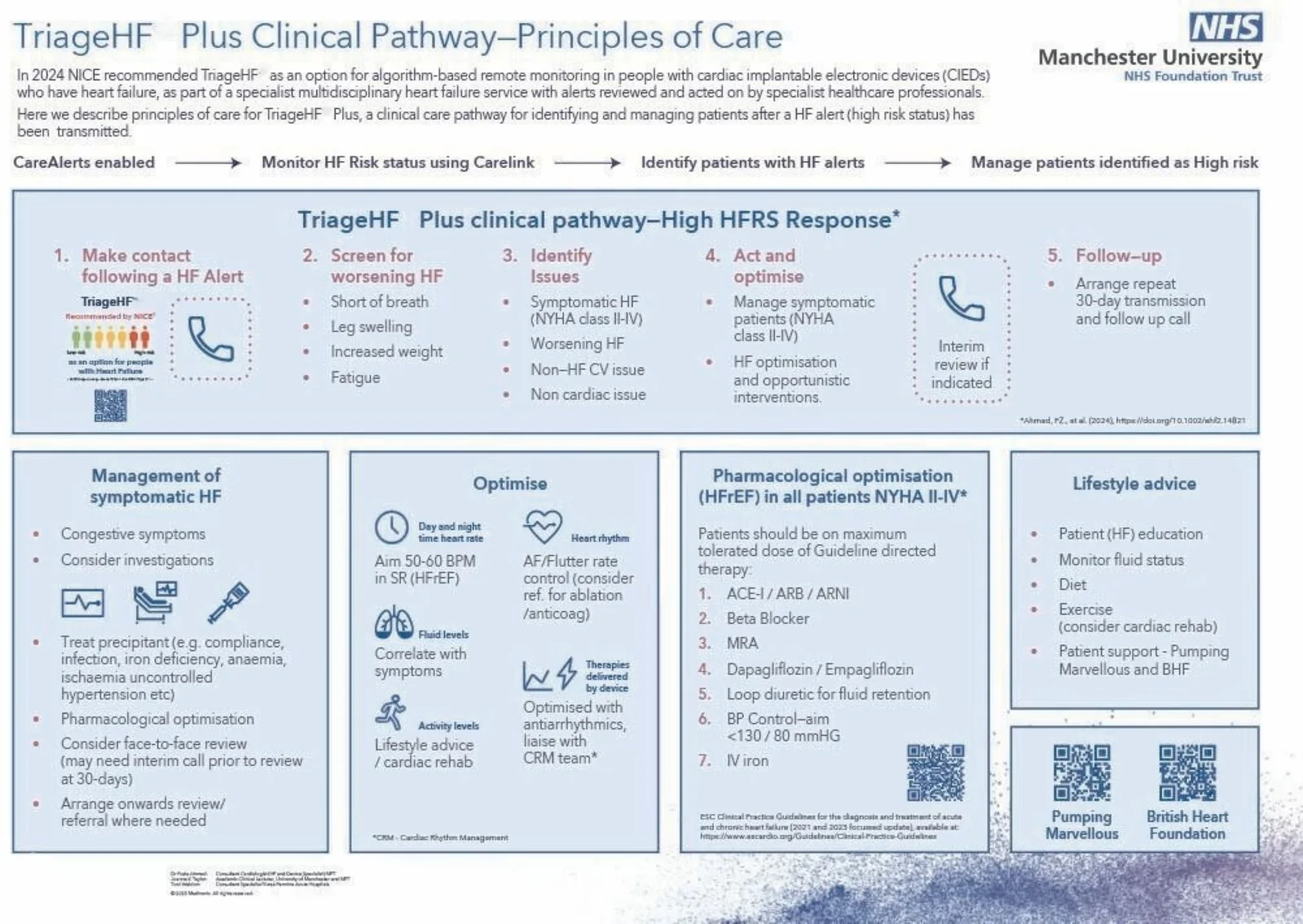
TriageHF Plus clinical pathway - principles of care poster. ACE, angiotensin-converting enzyme; ARB, angiotensin receptor blocker; ARNI, angiotensin receptor/neprilysin inhibitor; BHF, British Heart Foundation; BP, blood pressure; HF, heart failure; HFrEF, HF with reduced ejection fraction; HFRS, heart failure risk score; MRA, mineralocorticoid receptor antagonists; NHS, National Health Service; NICE, National Institute of Health and Care Excellence; NYHA, New York Heart Association, SR, sinus rhythm.

Patients with HF are often eligible for ‘complex’ cardiac implantable electronic devices (CIED), namely, cardiac resynchronisation therapy with pacemaker (CRT-P) CRT combined with a defibrillator (CRT-D) and implantable cardioverter-defibrillators (ICD).^6^ There are a variety of multi-parametric algorithm-based remote monitoring systems available, notably in the UK; TriageHF (Medtronic), HeartLogic (Boston Scientific) and HeartInsight (Biotronik). TriageHF is a HF algorithm which leverages routinely monitored health data from cardiac devices to calculate a patient’s risk of HF events in the next 30-days (low, medium, high; this has been described previously).^7–9^ The more abnormal the physiological parameters, the higher the risk status. ‘High’ risk status triggers a HF alert to be notified to the clinical team via Carelink. TriageHF Plus is a remote HF monitoring pathway for individuals with compatible CIEDs. As visually presented in figure 1, the pathway is triggered by a high-risk transmission (HF alert), signifying elevated risk of HF hospitalisation in the next 30 days.^9 10^ The remote monitoring platform is monitored by the HF team (usually an HF nurse) on a twice-weekly basis. After reviewing all HF alerts, patients are assessed using a structured phone-call based assessment. Clinical interventions thereafter are at the discretion of the assessor in line with guideline-recommended medical therapy. Prompts are provided to encourage proactive practice, commonly temporary up-titration of diuretics for congestive symptoms, optimisation of guideline-recommended medical therapies or arrangements for further investigations such as blood tests or echocardiogram or face-to-face clinical review (see figure 2). All HF alerts are followed up in 30days with both a repeat transmission and telephone assessment. A repeat 21-day transmission is scheduled for all medium-risk cases.^11^

In 2021, European Society of Cardiology HF guidelines did not recommend routine remote monitoring of HF diagnostics from cardiac devices due to lack of evidence of impact on clinical outcomes, such as hospital admissions and mortality.^12^ Since then, emerging evidence has changed the landscape of HF remote monitoring, and the subsequent 2023 HRS/EHRA/APHRS/LAHRS Expert Consensus Statement gave a 2 a recommendation to remotely monitor HF diagnostics.^13^ A recent pooled analysis of multi-sensor CIED-based remote monitoring reported reduced HF hospitalisations;^14^ however, only TriageHF and HeartLogic have been tested as part of a structured clinical care pathway for worsening HF.^15^ The TriageHF Plus Study (ClinicalTrials.gov Identifier NCT04177199), published in early 2024, demonstrated that using HF alerts as part of a remote patient management pathway (TriageHF Plus) reduced all-cause hospitalisations between 31–58% compared with usual care without adverse treatment-related effects.^16^ In response to new evidence, the United Kingdom (UK) National Institute of Health and Care Excellence (NICE) recommended HF alerts (specifically TriageHF Plus and HeartLogic) to be used as an option for people with HF and a compatible CIED in the UK in October 2024.^17–19^

Although the general practice of remote monitoring of CIEDs has been shown to be cost-effective, the economic value of a HF clinical care pathway driven by multiparametric HF alerts has not been evaluated.^20–22^

In this research, a health economic model was developed to conduct a cost-effectiveness analysis of the TriageHF Plus pathway using real-world data from the TriageHF Plus study.^16^

## Methods

### Study design, setting and participants

TriageHF Plus (NCT04177199) was a real-world, multicentre study of 758 participants across three National Health Service (NHS) sites in Greater Manchester, UK, including two cohorts with patients aged 18+ years, with an existing TriageHF compatible CIED capable of automated transmissions (Medtronic, Minneapolis, Minnesota, USA).^16^ Patients were recruited regardless of HF status; however, given the nature of the CIED population, the vast majority had a diagnosis of HF. Inclusion criteria were designed to represent a typical cohort if a hospital activated all devices to facilitate remote monitoring. The TriageHF Plus cohort (n=443) was actively recruited where, in addition to usual care, participants underwent remote monitoring following the TriageHF Plus clinical pathway after activation of device alerting capabilities. The time-matched ‘usual care’ cohort (n=315) was identified from hospital records as patients meeting study criteria but who did not undergo device alert activation. To be included in the analysis cohort, a minimum of 90 days of transmission data were required during the time horizon. Follow-up ran from September 2019 until February 2021, mean 433.6 days. Hereafter, this full-time horizon is synonymous with ‘total follow-up’. The primary outcome was the difference in unplanned, all-cause hospital admissions (hospitalisations). Prespecified minimum sample size was 450 patients followed up over at least 12 months; however, recruitment was expanded due to reduced hospitalisation rates during COVID-19.

Usual care was defined as compliance with national HF guidelines and usual CIED follow-up, but without action of HF alerts.^6 23^ Current NICE guidance recommends remote monitoring for people with HF; therefore, a scenario analysis was conducted for the 91.6% of the study population with HF. As the study spanned the COVID-19 pandemic, to examine the impact of the pandemic on results, a second scenario analysis was conducted using data from the pre-pandemic follow-up period, mean 173.0 days. Hereafter, this is called the ‘pre-COVID-19 follow-up’.^16 24^

Characteristics for study participants across the total follow-up and the two scenario analyses are presented in table 1.

**Table 1:** Patient characteristics of the TriageHF Plus study population

	Total follow-up (n=758)	Pre-COVID-19 follow-up (n=583)	HF population (n=694)
Age (mean (SD))	67.8 (12.7)	67.7 (13.1)	68.7 (11.7)
Sex (female) (N(%))	210 (27.7%)	171 (29.3%)	185 (26.7%)
Prior hospitalisation* (N(%))	144 (19.0%)	108 (18.5%)	132 (19.0%)
CIED type: CRT-D (N(%))	395 (52.1%)	314 (53.9%)	386 (55.6%)
CIED type: CRT-P (N(%))	222 (29.3%)	182 (31.2%)	193 (27.8%)
CIED type: ICD (N(%))	141 (18.6%)	87 (14.9%)	115 (16.6%)
NYHA status: no HF (N(%))	44 (5.8%)	32 (5.5%)	N/A
NYHA status: type I (N(%))	155 (20.4%)	106 (18.2%)	155 (22.3%)
NYHA status: type II (N(%))	328 (43.3%)	258 (44.3%)	328 (47.3%)
NYHA status: type III or IV (N(%))	211 (27.8%)	170 (29.2%)	211 (30.4%)
NYHA status: unknown (N(%))	20 (2.6%)	17 (2.9%)	N/A
LVEF >50% (N(%))	194 (25.6%)	159 (27.3%)	153 (22.0.%)
LVEF 35–50% (N(%))	287 (37.9%)	222 (38.1%)	275 (39.6%)
LVEF <35% (N(%))	268 (35.4%)	195 (33.4%)	260 (37.5%)
AF/atrial flutter (N(%))	369 (48.7%)	286 (49.1%)	340 (49.0%)
IHD (N(%))	436 (57.5%)	349 (59.9%)	420 (60.5%)
ACHD (N(%))	37 (4.9%)	30 (5.2%)	27 (3.9%)
Prior AV node ablation (N(%))	101 (13.3%)	81 (13.9%)	83 (12.0%)
Diabetes (N(%))	193 (25.5%)	149 (25.6%)	184 (26.5%)
CKD stage ≥3 (N(%))	283 (37.3%)	220 (37.7%)	281 (40.5%)

* At least one non-elective hospitalisation in the 6 months prior to enrolment.

ACHD, adult congenital heart disease; AF, atrial fibrillation; ARNI, angiotensin receptor neprolysin inhibitor; AV, atrioventricular; CIED, Cardiac implantable electronic device; CKD, chronic kidney disease; CRT-D, cardiac resynchronisation therapy with defibrillator; CRT-P, cardiac resynchronisation therapy with pacemaker; HF, heart failure; HFrEF, Heart failure with reduced ejection fraction; HFRS, Heart Failure Risk Score; ICD, implanted cardiac defibrillator; IHD, ischaemic heart disease; LVEF, left ventricular ejection fraction; NYHA, New York Heart Association functional classification; SR, sinus rhythm.

### Economic model structure

An economic model was conceptualised in accordance with the decision problem (table 2) and developed in adherence with the NICE reference case^25^ in Microsoft Excel (Microsoft Corporation, Washington, USA). The model had a two-state, regression-based structure which estimated the cost-effectiveness of TriageHF Plus compared with usual care. The cohort entered the model in the ‘alive’ state. A 1-month cycle length was required to capture the 30-day hospitalisation risk predicted by the TriageHF algorithm. In each cycle, a proportion of the cohort remained in the ‘alive’ state, and a proportion moved to the ‘dead’ health state. The proportion of the cohort in each health state at any point in time in the model was informed by survival analysis. The cohort accrued costs and benefits while in the ‘alive’ state over their lifetime. The ‘base case’/primary model was based on a typical hospital site—serving a population of 300 eligible patients for CIED remote monitoring. The probabilistic base case and key scenario analyses used published data from the TriageHF Plus study to inform the population characteristics, the per-month probability of a HF alert and the per-month hospitalisation event probability. Additional scenarios investigated the cost-effectiveness by CIED subgroup (CRT-P; CRT-D; ICD). These were informed by an R-based (http://www.r-project.org) post-hoc analysis of the individual patient-level data (IPD). These analyses used two covariate adjusted regression models to estimate the impact of TriageHF Plus on per-month probabilities of hospitalisations and HF alerts (covariables presented in table 1). A description of the IPD analysis methods and results is presented in the online supplemental file 1 (Section 1).

**Table 2:** Decision problem and pharmacoeconomic guidelines

Element	Decision
Model structure	Two-state (alive/dead) regression-based model
Populations	A cohort of people with TriageHF-compatible CIED
Scenarios	Population with a diagnosis of HF Pre -COVID-19 follow-up period Subgroups based on CIED (IPD only)
Intervention	TriageHF Plus
Comparator	Usual care
Outcomes	Incremental costs and quality-adjusted life years (QALYs) Incremental cost-effectiveness ratio Net monetary benefit Net health benefit
Perspective	England and Wales NHS and PSS
Cycle length	One month
Time horizon	Lifetime
Discount rates	3.5% for both costs and benefits
Cost-effectiveness threshold	£20 000 per QALY gained

CIED, cardiac implanted electronic device; HF, heart failure; IPD, individual patient data; NHB, net health benefit; NHS, National Health Service; PSS, personal social services.

Considered costs were NHS and personal social services. An annual discount rate of 3.5% was applied to both costs and benefits. Benefits were expressed as quality-adjusted life years (QALYs).

### Model parameters

Derivation of inputs for the economic model is described below in the methods section.

#### Hospitalisations

Regression models, using inverse probability of treatment weighting, were built to compare hospitalisation rates between cohorts. An annual hospitalisation rate of 0.58 in the usual care cohort was observed, equating to a per-month hospitalisation probability of 4.69%, and this was applied to the model. The incident rate ratios are provided in Section 1 of online supplemental file 1. The TriageHF Plus cohort hospitalisation rate was derived by applying the incidence rate ratios (IRRs) to the usual care cohort hospitalisation rate.

The estimated IRR in the CIED analysis across the total follow-up was 0.57 (95% CI 0.40 to 0.81). An exploratory, deterministic, breakeven analysis was conducted to determine the hospitalisation rate IRR at which TriageHF Plus was cost-neutral and cost-effective compared with usual care.

#### HF alerts

Per-month probability of a HF alert from the TriageHF Plus group was 2.91%. This probability was assumed to be equivalent for the pre-COVID-19 follow-up and the HF population scenario analysis and constant over the cohort’s lifetime. In the CIED scenario analyses, a logistic regression model estimated the probability of a HF alert. Regression equations and per-month probabilities for all scenario analyses are provided in online supplemental file 1 (Section 1).

#### Mortality

Six conventional parametric regression models were fit to IPD-based time-to-all-cause mortality data. The long-term predictive plausibility of each regression model, presented in the online supplemental file 1 (Section 2), was externally validated using published, peer-reviewed literature and clinical expert opinion. The Weibull model was used in the base case analyses. Mortality rates for the general population were sourced from life tables, compared with the estimates from the Weibull model and applied where appropriate. Due to a paucity of data, equivalent mortality estimates across both cohorts were assumed. The model employed a lifetime horizon (table 2). It was assumed life expectancy could not exceed 100 years. The total time horizon was 32.2 years (386 cycles).

#### Health-related quality of life

QALY gains were calculated from time spent by the cohort at a certain health-related quality of life (HRQoL). HRQoL is measured in utilities, a value of one represents perfect health and zero is equivalent to death. The model employed a multiplicative approach to calculate QALYs as per NICE guidance.^26^

Annual EuroQol five-dimensional questionnaire (EQ-5D-3L) utility estimates for each NYHA class were sourced from published literature.^27^ These were compared with UK-specific age-adjusted and sex-adjusted general population utility norms.^28^ The baseline population distribution across NYHA classes (table 1) was used to derive a weighted average annual utility decrement for the HF population (−0.109) and the weighted average per-event hospitalisation decrement (−0.070). This hospitalisation decrement was applied in the cycle the hospitalisation occurred (decrements provided in online supplemental file 1, section 3).

#### Costs and resource use

NHS-specific publicly available sources informed the costs in the model where possible (cost year 2022/2023). A summary of costs is presented in table 3; additional detailed breakdowns are provided in online supplemental file 1 (Section 2). Annual infrastructure costs included access to remote monitoring platforms, brief induction training when required and technical support. Staff training on using the pathway was minimal, as responses to an alert aligned with guideline-recommended management and clinicians retained the discretion to provide usual clinical care in response to HF alert (see figure 2).

**Table 3:** Costs in the model

	Scenarios			Source
	Base case	Pre-COVID-19 follow-up	HF population	
Hospitalisation costs				
All-cause hospitalisation cost (per event)	£2762	£2762	£2762	NHS England data using SUSHRG costing codes.
Annual Triage Plus infrastructure costs				
Annual infrastructure cost of TriageHF Plus per person.	£100	£100	£100	NICE DAP.^17^ Please note that this is equivalent regardless of site size.
Per-cycle monitoring costs for usual care and TriageHF Plus (without HF alerts) cohorts				
Professional cost	£96.91	£96.91	£96.91	Frequency informed by published literature. Costs were from the PSSRU and NHS reference costs.^14 15^
Investigation costs	£6.83	£6.83	£6.83	Frequency informed by published literature.^6 23^ Unit costs from NHS reference costs.^14^
Drug acquisition costs	£2.87	£2.90	£3.02	The posology and cost of ramipril (ACE inhibitor); carvedilol (beta blocker); spironolactone (MRA) and furosemide (diuretic) were extracted from BNF^20^ and eMIT.^20^ The percentage requiring each drug was informed by the TriageHF study.
Per-cycle monitoring costs for TriageHF Plus cohort (with HF alert)				
Drug acquisition costs	£3.32	£3.24	£3.38	The posology and cost of ramipril (ACE inhibitor); carvedilol (beta blocker); spironolactone (MRA) and furosemide (diuretic) were extracted from BNF^20^ and eMIT.^20^ The percentage requiring each drug was informed by the TriageHF study.
Cost of initial call (52.2%)	£81.00	£83.88	£81.00	The staff time required (by nurse band) per population was informed by the TriageHF study. Cost per staff time was informed by the PSSRU.^26^
Cost of follow-up calls (43.8%)	£69.79	£57.87	£71.59	
Cost of further investigations (7.1%)	£73.46	£73.46	£73.46	Frequency informed by the TRIAGE-HF study and assumptions. Unit costs from NHS reference costs.^14^
Cost of an outpatient cardiologist visit (10%)	£186.38	£186.38	£186.38	NHS reference costs.^14^

ACE, angiotensin-converting enzyme; BNF, British National Formulary; DAP, Diagnostics Assessment Programme; eMIT, UK Government Drugs and pharmaceutical electronic market information tool; HF, heart failure; MRA, mineralocorticoid receptor antagonists; NHS, National Health Service; NICE, National Institute of Health and Care Excellence; PSSRU, Personal Social Services Research Unit; SUSHRG, Secondary Uses Services Healthcare Resource Group.

#### Usual care monitoring and drug acquisition costs

Per-cycle clinical investigations and HF-related visits to medical professionals were based on national HF guidelines.^6 23^ Data from the TriageHF Plus study were used to determine the market share across the main drug classes available at the time of the study (table 3). The per-cycle resource and drug requirements were applied to NHS costs^14 15^ to derive monitoring and drug acquisition costs. These per-cycle monitoring costs were also accrued by those in the TriageHF Plus cohort without a HF alert.

#### TriageHF Plus resource use and costs

Infrastructure/remote patient management costs for TriageHF Plus were established at an annual cost of £100 per-person per-year.^17^

Additional resource costs associated with a HF alert were accrued in the cycle the HF alert occurred. The mean staff time requirements associated with each consultation (initial and 30-day follow-up calls) were captured. Interim consultations only occurred if clinically indicated and were not captured in the model.

It was assumed that those with an investigation would have three blood tests (full blood count, plasma creatinine and cardiac enzyme) plus one of either chest X-ray, ECG or echocardiogram. Healthcare utilisation (HCU) touchpoint analyses and expert opinion informed the percentage of the population who experienced a cardiology outpatient (OP) appointment (10% of the population with HF alerts).

#### HCU touchpoints

HCU touchpoints captured were hospitalisations, emergency department (ED) attendances and OP cardiology clinics. Lack of established guidance on costings processes prohibited the inclusion of ED attendances in the economic model. Instead, ED attendance rates and costings for TriageHF Plus and usual care were compared in a complementary analysis.

Costings for hospitalisations were obtained using Secondary Uses Services Healthcare Resource Group (SUSHRG) costing codes. Minimum requirements for episode cost extraction were admission date, discharge date and valid SUSHRG code. Where applicable, short stay emergency adjustments tariffs were applied to account for differences in length of stay. Where the SUSHRG codes lacked associated cost, two clinicians (JKT and FZA) selected the most clinically suitable alternative diagnosis using a conservative approach to admission complexity and comorbidity status. The median (IQR) in-trial cost of a hospitalisation was £2366 (£785–£4786) and £3140 (£933–£5036) for the TriageHF Plus and usual care cohorts, respectively. The total median (IQR) hospitalisation cost of £2762 (£826–£4846) was conservatively used to inform both cohorts in the model.

ED attendance and OP data were similarly obtained (detailed episode selection and costings provided in Section 4 of online supplemental file 1).

### Model outputs

Outcomes per person over a life-time horizon (32.2 years) are presented in the model results. A probabilistic sensitivity analysis (PSA) with 10 000 simulations was used in the base case to allow results to converge. The PSA captured and quantified uncertainties in the model parameters and assumptions. Each simulation used a different set of values for the uncertain parameters sampled from an appropriate statistical distribution, displayed in the online supplemental file 1 (Section 2).

The PSA estimated the mean (95% uncertainty intervals (UI)) lifetime costs and QALYs for TriageHF Plus and usual care. The 2.5th and 97.5% percentiles were used to estimate the uncertainty limits. Incremental cost and QALYs from each simulation are presented on the cost-effectiveness plane. These outcomes were compared against the NICE threshold of £20 000 per QALY gained to estimate the incremental cost-effectiveness ratio (ICER), net monetary benefit (NMB) and net health benefit.

#### Patient and public involvement

The TriageHF Plus study was reviewed by the Manchester University NHS Foundation Trust Cardiovascular Patient and Public Involvement panel prior to opening.

## Results

The entire study population had a mean age of 67.8 years, 27.7% were female and a mix of CIED types (52.1% CRT-D, 29.3% CRT-P and 18.6% ICD). Per-month hospitalisation probabilities for the TriageHF Plus cohort were 2.00% for the total follow-up and 2.56% for the pre-COVID-19 follow-up.

### Base case results

TriageHF Plus was found to be both dominant (increased QALYs and lower costs) and cost-effective (ICER<£20 000) in 99.4% of lifetime probabilistic simulations (presented in table 4), where a lifetime was found to be an average of 5.49 years (discounted) or 6.32 years (undiscounted) from study enrolment.

**Table 4:** Probabilistic base case and model results (mean, 95% UI)

Patient-level summary	TriageHF plus	Usual care	Incremental
Total average costs	£11 563 (£9629 to £13 987)	£15 551 (£14 560 to £16 619)	–£3989 (–£5563 to –£1812)
Total average QALYs	4.10 (3.77 to 4.32)	4.09 (3.76 to 4.31)	0.01 (0.00 to 0.01)
ICER (per QALY gained)			–£445 557
Probability of being cost-effective (threshold of £20 000 per QALY gained)			99.4%
Probability of being cost saving			99.4%
Incremental NMB			£4168 (£1909 to £5800)
Incremental NHB			0.21 (0.10 to 0.29)

ICER, incremental cost-effectiveness ratio; NHB, net health benefit; NMB, net monetary benefit; QALY, quality-adjusted life years; UI, uncertainty interval.

Lifetime total costs for a person in the TriageHF Plus cohort were estimated to be £11 563 (£9629–£13 987). This was £3989 (£1812–£5563) less than usual care (£15 551 (£14 560–£16 619)). The TriageHF Plus cohort improved HRQoL, with a gain of 0.01 (0.00–0.01) QALYs per person and was dominant over usual care, indicating it is cost-saving and cost-effective. Incremental NMB was estimated at £4168 (£1909–£5800) per QALY gained per lifetime. This finding was consistent across shorter time horizons, indicated by a positive NMB after 1 year and 5 years of monitoring, at £740 (£337–£1034) and £2885 (£1433–£4049), respectively (see table 5 below).

**Table 5:** Additional scenario analyses: TriageHF Plus versus usual care (2000 probabilistic iterations)

Parameter	Per-person outcomes: Mean (95% Credible Interval*)*			Incremental hospitalisations: Mean (95% UI)			
	Total incremental costs	ICER	NMB	Per-person	Site size: 50	Site size: 100	Site size: 300
Base-case parameters*	−£3989 (−£5563 to −£1812)	−£445 557 (dominating)	£4168 (£1909 to £5800)	−1.97 (−2.62 to −1.07)	−99 (−112 to −85)	−197 (−215 to −179)	−591 (−623 to −559)
1-year time horizon	−£707 (−£991 to −£318)	−£437 962 (dominating)	£740 (£337 to £1034)	−0.31 (−0.41 to −0.17)	−16 (−18 to −13)	−31 (−34 to −28)	−93 (−98 to −88)
5-year time horizon	−£2760 (−£3871 to −£1362)	−£441,500 (dominating)	£2,885 (£1433 to £4049)	−1.28 (−1.71 to −0.73)	−64 (−73 to −55)	−128 (−140 to −116)	−384 (−405 to −363)
Pre-COVID-19	−£2901 (−£4971 to −£130)	−£407,417 Dominating	£3,043 (£162 to £5199)	−1.54 (−2.39 to −0.37)	−77 (−87 to −67)	−154 (−168 to −140)	−462 (−486 to −438)
HF population	−£4220 (−£5858 to −£1876)	−£428 958 (dominating)	£4,417 (£1982 to £6118)	−2.11 (−2.79 to −1.11)	−106 (−119 to −92)	−211 (−231 to −192)	−633 (−667 to −599)
CRT-D subgroup	−£2277 (−£3946 to −£805)	−£405 501 (dominating)	£2,389 (£863 to £4119)	−1.24 (−1.93 to −0.63)	−62 (−71 to −53)	−124 (−137 to −111)	−372 (−394 to −350)
CRT-P subgroup	−£2410 (−£4361 to −£700)	−£404 581 (dominating)	£2,530 (£754 to £4562)	−1.31 (−2.13 to −0.60)	−66 (−75 to −56)	−131 (−145 to −117)	−393 (−417 to −369)
ICD subgroup	−£3441 (−£6412 to −£1066)	−£447 694 (dominating)	£3,595 (£1139 to £6685)	−1.69 (−2.92 to −0.70)	−85 (−97 to −72)	−169 (−187 to −151)	−507 (−538 to −476)

* Total follow-up, total population, lifetime horizon.

CRT-D, cardiac resynchronisation therapy with defibrillator; CRT-P, cardiac resynchronisation therapy with pacemaker; HF, heart failure; ICD, implanted cardiac defibrillator; ICER, incremental cost-effectiveness ratio; NMB, net monetary benefit; UI, uncertainty interval.

**Figure 3:**
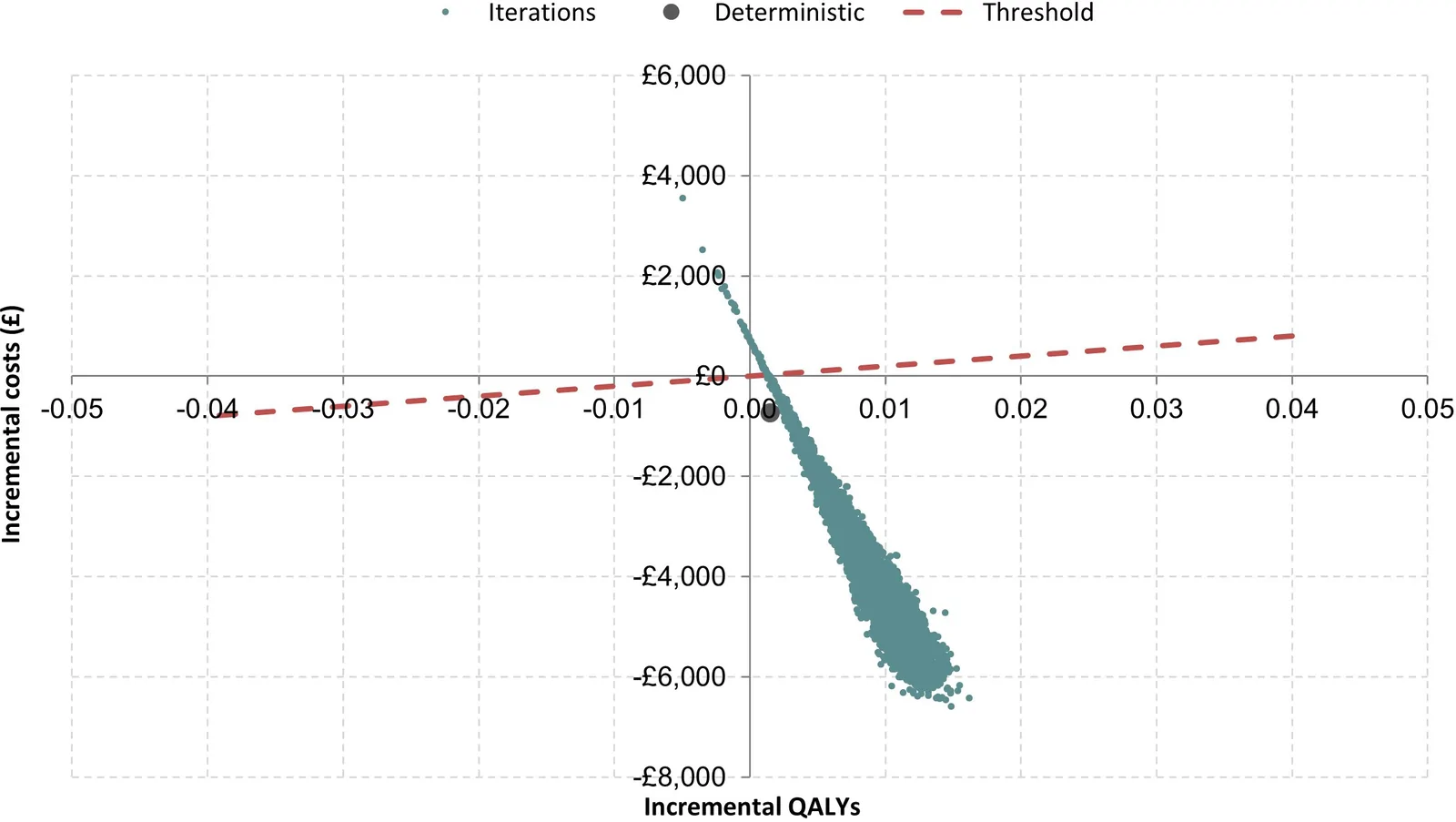
Cost-effectiveness plane. ICER, incremental cost-effectiveness ratio; QALY, quality-adjusted life-year.

The cost-effectiveness plane (figure 3) showed TriageHF Plus saved costs and accrued QALYs in 99.4% of the 10 000 probabilistic simulations, suggesting that TriageHF Plus is dominant compared with usual care. TriageHF Plus was also found to be cost-effective in 99.4% of simulations at a threshold of £20 000 per QALY gained. This finding was consistent across all thresholds of cost-effectiveness, indicated by the cost-effectiveness acceptability curve (online supplemental file 1, Section 5).

**Figure 4:**
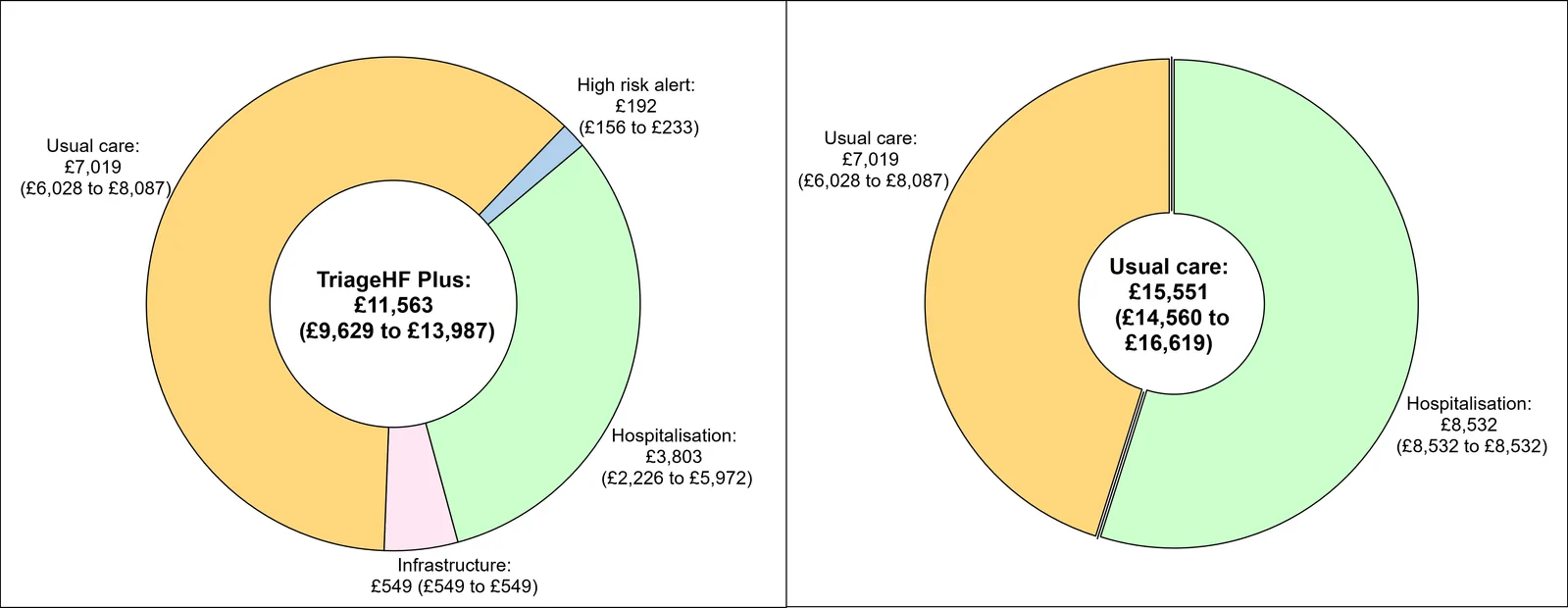
Breakdown of probabilistic costs for TriageHF Plus compared with usual care.

Within the TriageHF cohort, 6.4% of the lifetime healthcare costs were attributed to the pathway infrastructure and resource use costs. This increase in infrastructure cost was offset by the reduction in hospitalisations (1.97 per person). Lifetime hospitalisation costs are displayed in the donut chart (figure 4).

TriageHF Plus infrastructure cost was fixed at £100 per person. Benefits and cost savings estimated increases in proportion with site size. In the base case analysis, of a 300-patient population, TriageHF Plus was estimated to reduce the number of hospitalisations by 93 (88–98) in the first year with an NMB of £740 (£337–£1034) per person. This amounted to 591 (559–623) lifetime hospitalisations avoided for a 300-patient population. To avoid one hospitalisation, costing £2762 (£862–£4856) per event, 1.7 people would need lifetime access to TriageHF Plus.

### Scenario analysis results

**Figure 5:**
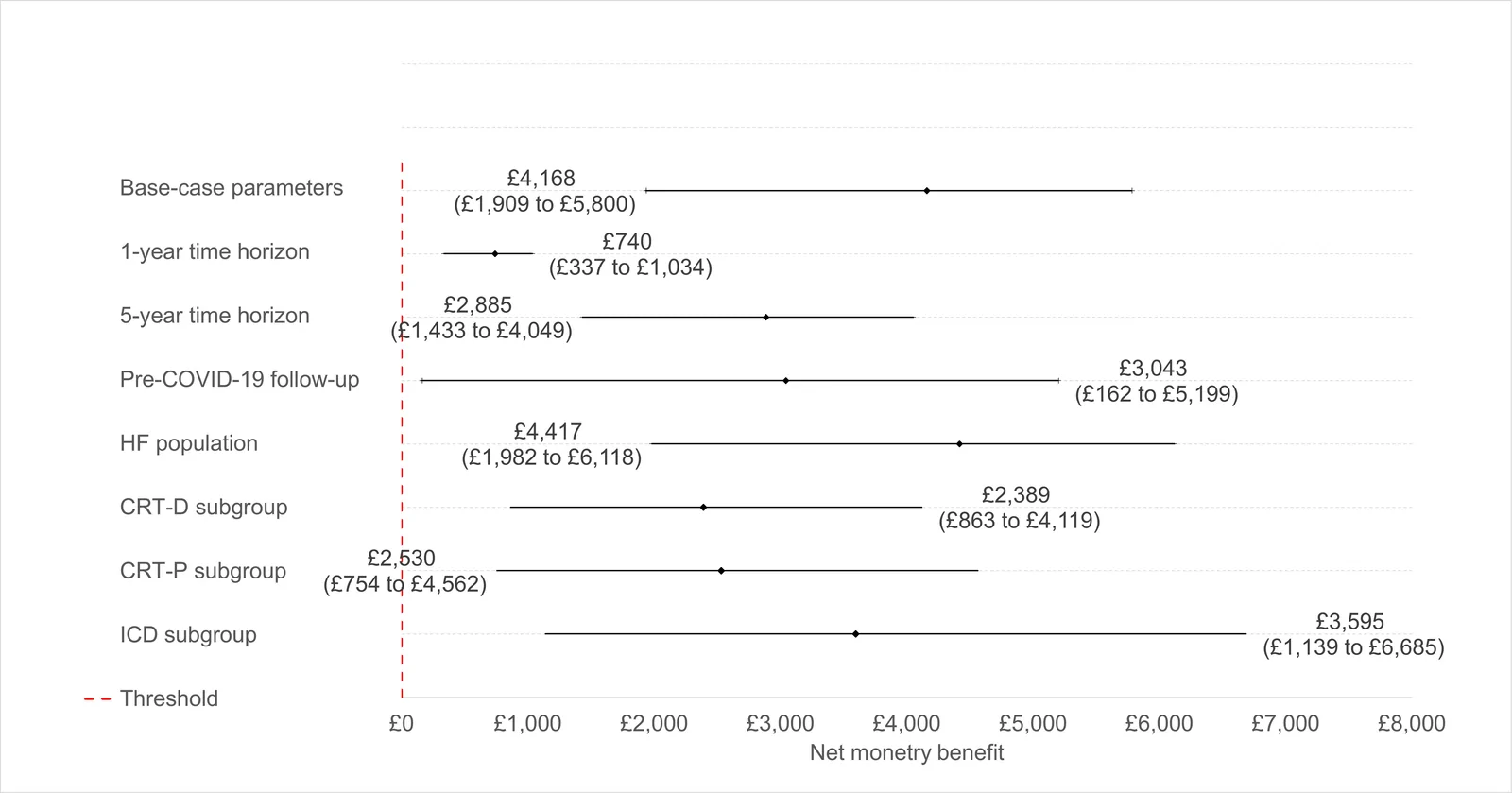
Net monetary benefit per person with TriageHF compared with usual care. CRT-D, cardiac resynchronisation therapy with a defibrillator; CRT-P, cardiac resynchronisation therapy device with a pacemaker; HF, heart failure; ICD, implantable cardioverter defibrillator.

Key results for the scenario analyses are presented in figure 5 and table 5. NMB remained positive in favour of TriageHF Plus across all scenarios. The greatest per-person lifetime cost-saving and NMB was observed in the HF population, £4417 (£1982–£6118); however, the effect of TriageHF Plus on NMB appeared to be consistent and cost effective regardless of baseline scenario.

Scenario analyses by CIED type used the regression equations derived from the IPD analysis to inform hospitalisation rate and probability of HF alerts. NMB remained positive across all scenarios, and incremental costs remained negative, consistent with the primary results (see figure 5 and table 5). The greatest cost savings and NMB were incurred in the ICD scenario; however, wide UIs suggested high variability.

### Exploratory breakeven analysis

In the base case, TriageHF Plus needed to reduce hospitalisation rates by 8.9% (IRR: 0.911) and 8.6% (IRR 0.914) to be cost-saving and cost-effective at a threshold of £20 000 per QALY, respectively. Using base-case parameters, NMB for an average 50-patient site was £208 400 (£194 643–£222 157) and £1 250 400 (£1 216 703–£1 284 097) for a 300-patient site. A full breakdown is provided in the online supplemental file 1 (Section 5).

### Complementary HCU analysis

A significant reduction in ED rates in the TriageHF Plus cohort compared with usual care was observed over the total follow-up, IRR 0.80 (0.64–1.00) p=0.046, and pre-COVID-19 follow-up, IRR 0.49 (0.29–0.82), p=0.007. Each ED visit was estimated to cost £165. See online supplemental file 1 (Section 6) for full breakdown.

A total of 4864 cardiology OP clinics occurred during total follow-up. After propensity score adjustment, there was a higher rate of clinics in the TriageHF Plus cohort across total follow-up; IRR 1.47 (1.39–1.56) p<0.001; but a lower rate in the pre-COVID-19 follow-up; IRR 0.82 (0.74–0.91) p<0.001. The impact on the rate of OP appointments is inconclusive.

## Discussion

In this prespecified analysis of the TriageHF Plus study, we report that utilisation of TriageHF Plus within the NHS had a 99.4% likelihood of being both cost-effective and cost-saving compared with current usual care. The key driver of cost savings was reduced hospitalisations. TriageHF Plus was estimated to save £3989 per person. Both small and large-sized sites were predicted to benefit from cost savings, with NMB ranging from £208 400 (£194 643–£222 157) for a 50-patient site, to £1 250 400 (£1 216 703–£1 284 097) for a 300-patient site. Adoption of TriageHF Plus could prevent 384 emergency hospitalisations over 5 years for a 300-participant site.

Breakeven analysis suggested TriageHF Plus only needed to reduce hospitalisation rates by 8.6% to be cost-effective, well short of the 43–58% reduction observed in the TriageHF Plus study. The HF subgroup, which accounted for 92% of the population studied, was associated with greatest cost savings, as expected given the intervention primarily focuses on optimising HF care and managing periods of disease instability. This said, TriageHF Plus was found to be cost effective in all scenario analyses and subpopulations, and thus a simple non-restrictive approach to patient selection would be reasonable.

This is the first paper to report on the cost-effectiveness of a device-based remote monitoring clinical pathway driven by multiparametric HF alerts. Both TriageHF and HeartLogic are now recommended by NICE as an option for algorithm-based remote monitoring in people with cardiac devices who have HF.^19^ Direct comparison of algorithm performance and efficacy is challenging due to differences in populations and outcomes assessed. Although there are differences in the physiological parameters assessed, there remains significant overlap.^15^ TriageHF Plus offers a major advantage to other systems as it is available across CRT-P, CRT-D and ICD devices and is backwards compatible with many legacy devices.

This analysis comes at a time of great need for cost-effective approaches to delivering HF care. Over the past three decades, the number of hospital beds in the UK has more than halved and continues to decline, a pattern replicated across many global healthcare systems.^29^ This reduction poses a challenge for HF care, a leading cause of bed occupancy in public health systems. Despite medication and treatment advancements for HF, it is projected that there will be 50% more hospitalisations due to HF by 2035 than in 2010.^30^ The reality of these projections and trends in disease burden highlights the need for innovative care models. In 2024, the NHS set out a vision to shift from analogue to digital, from sickness to prevention and from hospital to community.^31^ Although a vision for the NHS, the strategy is universally relevant. Our findings support upfront investment in resources to build capacity in remote monitoring pathways such as TriageHF Plus to benefit both patient and the NHS. This paper, along with the recently published NICE review, would also support the adoption of device-based HF remote monitoring across UK hospital sites.

For patients with CIEDs, remote monitoring technologies can promote safe and effective HF management when integrated into a structured clinical pathway. HF alerts are now recommended as an option for HF patients with a device by NICE.^17^ The infrastructure to facilitate nationwide adoption of the TriageHF Plus pathway is well-established, the only limitation being clinical staffing and re-organisation of workflows. The TriageHF Plus pathway was designed to be executed by HF specialist nurses; however, with the increasing use of devices to manage HF in the NHS and national shortage of specialist nurses, upskilling of arrhythmia nurses and cardiac physiologists offers a viable alternative for resourcing. New sites may choose to adapt the pathway to their local needs, considering key variables such as geography, local provision of services and staff experience.

Considering the generalisability of cost-savings observed in this analysis to other countries, extrapolation of the magnitude of effect on outcomes and cost-effectiveness should be approached with caution. While published data on HF hospitalisation risk reduction are consistent,^2 14 32^ factors such as differing healthcare structures, reimbursement models and population risk are likely to impact on the magnitude of savings which can be expected. Nevertheless, the potential for comparable cost savings in other healthcare systems is great, and expansion of infrastructure is relatively straightforward given this is already operational. The main barrier to implementation observed in the UK was the adaptation of working practices within the HF team to receiving HF alerts from the remote monitoring platforms. However, once embedded, most teams opted to retain the practice beyond the evaluation period.^33^

### Limitations

The TriageHF Plus study involved three hospitals across Greater Manchester, a relatively urban geographical area of the UK; therefore, generalisability to other areas would need consideration. The study population may also not be generalisable to all patients with eligible CRT/ICD devices as there was a tendency to recruit patients with a confirmed HF diagnosis, while very frail individuals and those with language barriers may be under-represented. This was a real-world time-matched cohort design, using propensity matching for comparative analysis, which is considered less robust than a randomised control design. The study follow-up period was impacted by the COVID-19 pandemic,^16 24^ hence inclusion of the pre-COVID-19 scenario analysis to partially mitigate this.

Various assumptions were made to run the economic model due to lack of data availability. Equivalent mortality was applied to both cohorts, based on lack of evidence to demonstrate otherwise. It was assumed the probability of hospitalisation remained constant over a patient’s lifetime, which is unlikely given older patients are more likely to be hospitalised. The impact of HF events on an individual patient’s subsequent long-term risk was not captured and therefore assumed to be nil. This is also unlikely to be true given the detrimental impact of hospitalisations, and both assumptions could be explored in future analyses utilising a larger sample size. These conservative assumptions may have led to an underestimation of the impact observed. Mean utilities for the small non-HF device population were assumed to be equivalent to NYHA class I; therefore, the utility decrements may be marginally overestimated. Statistical assumptions related to survival modelling may have had a confounding effect on the results. Additionally, although both cohorts were similarly affected by stay-at-home guidance during the COVID-19 pandemic, current-era hospitalisation rates are expected to be higher, potentially altering the magnitude of benefit and cost savings. An analysis of contemporaneous data is currently underway to explore this further.

Attempts to provide accurate costings were made; however, where data were lacking, conservative assumptions were used. It was assumed HRQoL utility decrement applied only to the month a hospitalisation occurred in, where the real-life impact of a hospitalisation may be much longer.^1^ The cost of ED attendances was not included in the model due to lack of robust costing methods; this was unlikely to significantly impact results given small costs observed (approximately £18.05 per patient per year). Costings in this model were based on published NHS reference costings. Enumeration costs and the £100 per patient flat cost of TriageHF Plus may differ from country to country. Future microcosting studies could be undertaken to enhance accuracy.

## Conclusion

The TriageHF Plus pathway is cost-effective for the remote monitoring of HF in patients with CIEDs. These economic benefits support the case for adding this remote-based monitoring pathway to standard care for eligible CIED recipients.

## Supplementary material

10.1136/heartjnl-2025-326702 online supplemental file 1

10.1136/heartjnl-2025-326702 online supplemental file 2

## Data Availability

Data are available upon reasonable request. All data relevant to the study are included in the article or uploaded as supplementary information.
