# Supplementary material for: Economic evaluation of the TriageHF Plus clinical pathway for device-based remote monitoring in heart failure
Source: Heart. 2025 Dec 17;112(18):e326702. doi: 10.1136/heartjnl-2025-326702 (PMC13539856; doi:10.1136/heartjnl-2025-326702)
Supplement: Supplementary file 1 [file heartjnl-112-18-s001.docx]

SUPPLEMENTARY MATERIAL

*A Health Economic Evaluation of the TriageHF Plus Clinical Care Pathway for the Device-base Remote Monitoring of Heart Failure. Fozia Z Ahmed and Rachel Harwood, David Lanctin, Sarah Medland, Daniela Afonso, S Mealing, Toni Weldon, Callum Blunt, Niall G Campbell, J K Taylor. 2025.*

# Glossary

| **Acronym** | **Meaning** |
| --- | --- |
| ACE-i | Angiotensin-Converting Enzyme Inhibitors |
| ACHD | Adult Congenital Heart Disease |
| ADMIDATE | Date of admission to hospital |
| ADMIMETH | Method of admission into hospital |
| AF Flutter | Atrial Fibrillation/Flutter |
| AIC | Akaike Information Criterion |
| APC | Admitted Patient Care |
| APPDATE | Appointment Date |
| ARB | Angiotensin II Receptor Blockers |
| ARNI | Angiotensin Receptor Neprilysin Inhibitor |
| ARRIVAL_DATE | Date of Arrival to Emergency Department |
| ARRIVAL_TIME | Time of Arrival to Emergency Department |
| BIC | Bayesian Information Criterion |
| CC | Comorbidity Code |
| CIED | Cardiac implantable electronic device |
| CKD | Chronic Kidney Disease Stage 3+ |
| CPRD | Clinical Practice Research Datalink |
| CRT-D | Cardiac Resynchronisation Therapy Device with a Defibrillator |
| CRT-P | Cardiac Resynchronisation Therapy Device with Pacemaker |
| DISDATE | Date of discharge from hospital |
| ECG | Electrocardiogram |
| ED | Emergency Department |
| EL | Elective Inpatient |
| e-MIT | Electronic Market Information |
| EMS | Electric Monitoring Service |
| GLMM | Generalised Linear Mixed Model |
| GP | General Practitioner |
| HF | Heart Failure |
| HFRS | Heart Failure Risk Score |
| ICD | Implantable cardioverter-defibrillator |
| ICD10 | International Classification of Disease 10^th^ Revision |
| IPD | Individual Patient Data |
| LVEF | Left-Ventricular Ejection Fraction |
| MAINSPEF | Department of outpatient appointment |
| MRA | Mineralocorticoid Receptor Antagonist |
| NEL | Non-Elective Long Stay |
| NES | Non-Elective Short Stay |
| NHS | National Health Service |
| NICE | National Institute for Health and Care Excellence |
| NYHA | New York Heart Association Functional Status |
| OP | Outpatient |
| SSEM | Short Stay Emergency Adjustments |
| STUDY_ID | Unique Identifier |
| SUSHRG | Secondary Uses Services Healthcare Resource Group |

# Section 1. Individual Patient Data Analysis

## Hospitalisation rates

### Methods

IPD analysis was performed in order to estimate the effects of cardiac device type. The CIED types included in this study were CRT-D, ICD, and CRT-P. The number of all-cause hospitalisations the patient experienced in their follow up window were modelled with a generalised linear mixed model with a negative binomial distribution and log link. The variables included are shown in table S1

The exposure time was calculated starting from enrolment (TriageHF Plus Cohort) or identified retrospectively (Usual Care Cohort). The final point to calculate exposure time to was either death, general censoring (e.g. lost to follow up) or the end of the study. The count data was modelled as a function of the natural log of exposure time (in months) as an offset variable to derive a rate per month rather than a count (this is outlined in equation 1). Random effects were applied to each individual to account for unobserved variables, the random effects were assumed to follow the normal distribution based on the central limit theorem. Exposure time was defined as the length of time that an individual in the study is at risk of hospitalisation.

Equation 1: The form of the fixed effects part of count-based model with a log link

$$\boldsymbol{log}\left( \boldsymbol{Hosp}_{\boldsymbol{Count}} \right)\boldsymbol{=}\boldsymbol{Intercept+\ldots}\boldsymbol{B}_{\boldsymbol{n}}\boldsymbol{TriageHF}_{\boldsymbol{i}}\boldsymbol{+ +offset(}\log\left( \boldsymbol{ExposTime}_{\boldsymbol{i}} \right)\boldsymbol{)}$$

Where i represents the i^th^ individual in the dataset.

### Results

Demographics of the cohort are presented here in Table S1

|  |  | Triage HF Plus Cohort (N=443) | Usual Care Cohort (N=315) | Total Cohort (N= 758) |
| --- | --- | --- | --- | --- |
| Age, mean(sd) | | 68.8 (11.2) | 66.2 (14.5) | 67.8 (12.7) |
| Sex | Male | 343 (77.4%) | 205 (65.1%) | 548 (72.3%) |
|  | Female | 100 (22.6%) | 110 (34.9%) | 210 (27.7%) |
| Prior Hospitalisation between March 2019 and September 2019 | Yes | 86 (19.4%) | 58 (18.4%) | 144 (19.0%) |
| Device Type | CRT-D | 259 (58.5%) | 136 (43.2%) | 395 (52.1%) |
|  | CRT-P | 79 (17.8%) | 143 (45.4%) | 222 (29.3%) |
|  | ICD | 105 (23.7%) | 36 (11.4%) | 141 (18.6%) |
| NYHA | No Heart Failure | 21 (4.7%) | 23 (7.3%) | 44 (5.8%) |
|  | I | 113 (25.5%) | 42 (13.3%) | 155 (20.4%) |
|  | II | 204 (46.0%) | 124 (39.4%) | 328 (43.3%) |
|  | III or IV | 100 (22.6%) | 111(35.2%) | 211 (27.8%) |
|  | Missing | 5 (1.1%) | 15 (4.8%) | 20 (2.6%) |
| Beta Blocker | Yes | 397 (89.6%) | 250 (79.4%) | 647 (85.4%) |
|  | Missing | 0 | 28 (8.9%) | 28 (3.7%) |
| ACE/ARB/ARNi | Yes | 338 (76.3%) | 217 (68.9%) | 555 (73.2%) |
|  | Missing | 0 | 29 (9.2%) | 29 (3.8%) |
| Diuretic | Yes | 219 (49.4%) | 155 (49.2%) | 374 (49.3%) |
|  | Missing | 0 | 30 (9.5%) | 30 (4.0%) |
| MRA | Yes | 218 (49.2%) | 116 (36.8%) | 334 (44.1%) |
|  | Missing | 0 | 31 (9.8%) | 31 (4.1%) |
| Current AF/ AF Flutter | Yes | 208 (47.0%) | 161 (51.1%) | 369 (48.7%) |
|  | Missing | 0 | <5 (<1.6%) | <5 (<0.7%) |
| IHD | Yes | 240 (54.2%) | 196 (62.2%) | 436 (57.5%) |
|  | Missing | 14 (3.2%) | 8 (2.5%) | 22 (2.9%) |
| ACHD | Yes | 11 (2.5%) | 26 (8.3%) | 37 (4.9%) |
|  | Missing | 6 (1.4%) | 0 | 6 (0.8%) |
| Ablation | Yes | 41 (9.3%) | 60 (19.0%) | 101 (13.3%) |
|  | Missing | 9 (2.0%) | <5 (<1.6%) | 12 (1.6%) |
| Diabetes | Yes | 111 (25.1%) | 82 (26.0%) | 193 (25.5%) |
|  | Missing | <5 (<1.1%) | 9 (2.9%) | 13 (1.7%) |
| CKD (stage 3+) | Yes | 180 (40.6%) | 103 (32.7%) | 283 (37.3%) |
|  | Missing | 10 (2.3%) | <5 (<1.6%) | 14 (1.8%) |
| LVEF | <35% | 180 (40.6%) | 88 (27.9%) | 268 (35.4%) |
|  | 35%≤LVEF<50% | 166 (37.5%) | 121 (38.4%) | 287 (37.9%) |
|  | ≥50% | 93 (21.0%) | 101 (32.1%) | 194 (25.6%) |
|  | Missing | <5 (<1.1%) | 5 (1.6%) | 9 (1.2%) |

*HF=Heart Failure, NYHA = New York Heart Association Functional Status, CRTP = cardiac resynchronisation therapy device with pacemaker, ICD = implanted cardiac defibrillator, CRT-D cardiac resynchronisation therapy device with a defibrillator , LVEF = left-ventricular ejection fraction, AF Flutter = atrial fibrillation/flutter, CKD = chronic kidney disease, IHD = ischaemic heart disease, ACHD = adult congenital heart disease, MRA= mineralocorticoid receptor antagonist, ACE-i= Angiotensin-converting enzyme inhibitors, ARB= angiotensin II receptor blockers, ARNI= angiotensin receptor- neprilysin inhibitor*

Resulting estimated rates of hospitalisation are shown in **Table S2**.

Table S2: Results of the IPD analysis estimating rate of hospitalisations

| Covariable | Total Cohort (total follow-up) | Total Cohort (Pre-COVID-19 follow-up) | Cohort with HF (total follow-up) |
| --- | --- | --- | --- |
| Intercept | **-8.413** | **-7.653** | **-8.783** |
| Age | 0.010 | -9.884e-05 | 0.013 |
| Male | 0.231 | 0.142 | 0.195 |
| NYHA I | -0.185 | -0.556 | Control |
| NYHA II | 0.440 | 0.157 | **0.610** |
| NYHA III or IV | **0.990** | 0.703 | **1.152** |
| CRT-P | -0.066 | -0.418 | -0.071 |
| ICD | 0.231 | -0.544 | 0.223 |
| LVEF 35-50 | 0.220 | -0.100 | 0.254 |
| LVEF <35 | 0.057 | -0.100 | 0.088 |
| AF Flutter Yes | 0.249 | **0.807** | 0.253 |
| CKD stage 3 or greater | 0.181 | 0.406 | 0.146 |
| IHD Yes | 0.172 | 0.245 | 0.148 |
| Diabetes Yes | 0.262 | -0.006 | 0.275 |
| ACHD Yes | -0.500 | 0.449 | -0.923 |
| Ablation Yes | -0.227 | -0.390 | -0.277 |
| Prior Hospitalisation (March 2019 to Sept 2019) | **0.721** | **0.769** | **0.734** |
| Treatment Group | **-0.562** | **-0.564** | **-0.521** |
| Log (ExposTime) | 1 | 1 | 1 |
| **Fit Statistics** | | | |
| R Squared | 0.180 | 0.180 | 0.176 |
| AIC | 1306.4 | 572.8 | 1261.5 |

*HF=Heart Failure, NYHA = New York Heart Association Functional Status, CRTP = cardiac resynchronisation therapy device with pacemaker, ICD = implanted cardiac defibrillator, LVEF = left-ventricular ejection fraction, AF Flutter = atrial fibrillation/flutter, CKD = chronic kidney disease, IHD = ischaemic heart disease, ACHD = adult congenital heart disease*

The base cases for the total cohort (total follow-up) and total cohort (pre-COVID-19 follow up) were as follows – Female, NYHA-No heart failure, Device Type- CRTD, LVEF >50%, Treatment Group- Control group. The base cases for the cohort with HF (total follow up) were as follows Sex- Female, NYHA-I, Device Type- CRTD, LVEF >50%, Treatment Group- Control group.

## HF alert probabilities

### Methods

The monthly probability of a person with TriageHF Plus sending a HF alert was used in the economic model to estimate the size of the cohort accruing additional costs. A logistic regression model was used to estimate this outcome.

A unique patient ID was captured within the model as a random effect to take into account the repeated measure nature of the outcome (i.e. probability of a HF alert was collected at multiple time points per individual).

Equation 2.1 outlines the form of the fixed effects part of logistic regression model for the probability of flagging as “high-risk” to the CareLink network.

Equation 2.1: The form of the fixed effects part of logistic regression model

$$\log it\left( P\left( HighRisk=1 | X \right) \right)=Intercept +\ldots+B_{n}\Pr iorHFHosp_{i,t}$$

Where i represents the i^th^ individual in the dataset and t is the timepoint of the observation.

Transmissions could be scheduled, manual, or alert. The transmission with the highest HFRS was taken in the case where there was more than one transmission per month. Where a transmission was not received in a month it was assumed that there was no high alert. The family used to model the data was binomial.

### Results

Estimates of HF alert rates are shown in **Table S3.**

Table S3: Results of the IPD analysis estimating rate of HF alerts

| Covariable | Total Cohort (total follow-up) | Total Cohort (Pre-COVID-19 follow-up) | Cohort with HF (total follow-up) |
| --- | --- | --- | --- |
| Intercept | **-3.599** | -13.767 | **-3.296** |
| Age | -0.009 | 0.027 | -0.007 |
| Male | **-0.879** | -0.529 | **-0.884** |
| NYHA I | 0.358 | 8.628 | Control. |
| NYHA II | 0.345 | 8.483 | -0.006 |
| NYHA III or IV | 1.014 | 8.990 | 0.674 |
| CRT-P | -0.264 | -0.324 | -0.163 |
| ICD | **-1.062** | -0.937 | **-0.959** |
| LVEF 35-50 | 0.232 | -0.613 | 0.174 |
| LVEF <35 | 0.247 | -0.133 | 0.196 |
| AF Flutter Yes | 0.317 | -0.636 | 0.263 |
| CKD stage 3 or greater | **0.853** | 0.452 | **0.802** |
| IHD Yes | 0.000 | -0.097 | 0.018 |
| Diabetes Yes | 0.227 | 0.211 | 0.243 |
| ACHD Yes | 0.205 | 0.569 | -0.006 |
| Ablation Yes | -0.064 | 0.456 | -0.341 |
| Prior Hospitalisation (March 2019 to Sept 2019) | 0.491 | 0.120 | 0.498 |
| **Fit Statistics** | | | |
| AIC | 2312.3 | 623.1 | 2268.5 |
| BIC | 2430.6 | 718.5 | 2379.6 |
| Random Effects | | | |
| Variance | 2.85 | 3.60 | 2.80 |
| Standard Deviation | 1.688 | 1.897 | 1.672 |

*HF=Heart Failure, NYHA= New York Heart Association functional status, CRTP = cardiac resynchronisation therapy device with pacemaker, ICD = implanted cardiac defibrillator, LVEF = left-ventricular ejection fraction, AF Flutter = atrial fibrillation/flutter, CKD = chronic kidney disease, IHD = ischaemic heart disease, ACHD = adult congenital heart disease*

The base cases for the total cohort (total follow-up) and total cohort (pre-COVID-19 follow up) were as follows – Female, NYHA-No heart failure, Device Type- CRTD, LVEF >50%. The base cases for the cohort with HF (total follow up) were as follows Gender- Female, NYHA-I, Device Type- CRTD, LVEF >50%.

## Breakdown by CIED type

**Table S4** summarises the CIED subgroup IPD analysis inputs, compared with the main analysis

Table S4: Summary of inputs in the exploratory IPD analysis compared with the main analysis.

| **Variable (all lifetime horizon)** | **Per-month probability of an all-cause hospitalisation event** | | | **Per-month probability of a high alert** | **Minimum IRR required (breakeven analysis)** | |
| --- | --- | --- | --- | --- | --- | --- |
|  | **Usual care** | **TriageHF plus** | **Incidence rate ratio (IRR)** |  | **Cost-effective** | **Cost-neutral** |
| **TriageHF Plus study data using inverse propensity matching** | | | | | | |
| Total follow-up in the total population (base-case) | 4.69% | 2.00% | 0.420 | 2.91% | 0.914 | 0.911 |
| Pre-COVID-19 follow-up in the total population | 4.69% | 2.56% | 0.540 | 2.91% | 0.911 | 0.907 |
| HF population | 4.69% | 2.00% | 0.420 | 2.91% | 0.914 | 0.911 |
| **IPD analysis** | | | | | | |
| CRT-D subgroup | 4.06% | 2.33% | 0.570 | 2.82% | 0.902 | 0.899 |
| CRT-P subgroup | 3.80% | 2.19% | 0.570 | 2.18% | 0.902 | 0.898 |
| ICD subgroup | 5.08% | 2.93% | 0.570 | 0.99% | 0.934 | 0.932 |

*IRR= Incidence rate ratio, HF=Heart Failure, CRT-D= cardiac resynchronisation therapy with a defibrillator, CRT-P Cardiac resynchronisation therapy device with a pacemaker, ICD= Implantable cardioverter defibrillator, IPD= Individual Patient Data*

# Section 2. Economic modelling: additional information

### Parameter distribution

To model uncertainty in the cost effectiveness model, parameters were assigned a distribution which are outlined in **Table S5**. Parameter distributions were aligned with those outlined in Briggs et al (2006) [1].

In order to account for second-order uncertainty around the appropriate parameter values, the model included a PSA. In the UK, NICE requests that all submissions include PSA, to provide an estimate of confidence around the model’s findings, and that the base case is presented probabilistically.

In the PSA, uncertainty parameters in the model were each sampled from an appropriate distribution, fitted using the mean and SE associated with the uncertainty parameter. In cases where the SE was unknown, it was assumed to be 10% of the mean value. Information around which distribution was used for each parameter within the model is provided in Table S4. The distributions were selected based upon recommendations for best practice based upon Briggs et al (2011).

For probabilities and utilities, beta distributions were used, while resource use paramaters are fitted with gamma distributions beta distributions are bound between the values of 0 and 1, whereas gamma distributions produce non-negative values). The distributions for each parameter are presented in uncertainty around estimates provided by the regression equations were incorporated into the model utilising the Cholesky matrix derived from the regression variance-covariance matrix.

Table S5: The distributions for the parameters included in the probabilistic model

| **Parameter** | **Distribution** |
| --- | --- |
| **Efficacy (published parameters only)** | |
| Usual Care v.s TriageHF IRR | Lognormal |
| Total study set v.s. pre COVID-19 set IRR | Lognormal |
| TriageHF Plus probability of high-risk flag (%) | Beta [0,1] |
| **Resource use (Low risk / Usual Care)** | |
| Percentage of population on pharmacological treatments (either beta blockers; ACE-I; diuretics, or MRA) (%) | Beta [0,1] |
| Frequency of GP visits (N per month) | Gamma |
| Frequency of cardiologist visit (N per month) | Gamma |
| Frequency of other physician visit (N per month) | Gamma |
| Frequency of Accident and emergency visit (N per month) | Gamma |
| Frequency of plasma creatine/ blood urea nitrogen tests (N per month) | Gamma |
| Frequency of blood count tests (N per month) | Gamma |
| Frequency of ECGs (N per month) | Gamma |
| Frequency of chest x-ray (N per month) | Gamma |
| Frequency of echocardiography (N per month) | Gamma |
| Frequency of cardiac enzyme test (N per month) | Gamma |
| **Resource use (high-risk flag)** | |
| Percentage of population on pharmacological treatments (either beta blockers; ACE-I; diuretics, or MRA) (%) | Beta [0,1] |
| Percentage who receive an initial consultation (%) | Beta [0,1] |
| Percentage who receive follow-up consultations (%) | Beta [0,1] |
| Average nurse time per band per initial consultation (minutes) | Gamma |
| Average nurse time per band per follow-up consultation (minutes) | Gamma |
| Proportion of people receiving further investigations (%) | Beta [0,1] |
| Frequency of plasma creatine/ blood urea nitrogen tests (N per 30-day monitoring period) | Gamma |
| Frequency of blood count tests (N per 30-day monitoring period) | Gamma |
| Frequency of ECGs (N per 30-day monitoring period) | Gamma |
| Frequency of chest x-ray (N per 30-day monitoring period) | Gamma |
| Frequency of echocardiography (N per 30-day monitoring period) | Gamma |
| Frequency of cardiac enzyme test (N per 30-day monitoring period) | Gamma |
| **Utilities** | |
| Utility by NYHA class (either I; II; III; IV, or no diagnosis) | Beta [0,1] |
| Hospitalisation utility decrement by NYHA class (either I; II; III; IV, or no diagnosis) | Beta [0,1] |
| TriageHF utility increment | Beta [0,1] |

IRR= Incidence Ratio Rate, *ACE-i= Angiotensin-converting enzyme inhibitors, MRA= mineralocorticoid receptor antagonist, GP=General Practitioner, ECG= Electrocardiogram, NYHA= New York Heart Association Functional Classification.*

### Survival Analysis

Survival was modelled for the TriageHF Plus population. There were a total of 19 deaths in the total follow-up period for the total population. Comparatively, there were a total of 18 deaths in the total follow-up period for the HF population. This only includes deaths prior to the end of the window.  Several models of survival were explored to determine the one that most accurately reflects real world survival of the target group for TriageHF Plus (**Table S6**).

Given that there were a relatively small number of deaths in the TriageHF Plus study, and, at the end of the clinical, 80% of participants were still alive. Therefore, the KM data informing the models were immature, with a large drop of patients at the end of the trial as patients were censored. This led to long-term extrapolations that may not reflect what would occur in a clinical setting. Indeed, the models that best fit the KM data (according to the goodness-of-fit statistics, namely AIC and BIC) were the Gompertz and the Weibull models. However, both these models estimated all people would be dead within 5 years. Given 26% of the population survived 10 years in a study conducted by Taylor et al. (2019) {Taylor, 2019 #76}, it is clear these models lack external validity. Indeed, given that the average age of the population in the study conducted by Taylor et al. (2019) was 77 years, which is ten years older than the population in the TriageHF Plus study, it was expected that the survival estimates from the OS regression models would exceed those reported by Taylor et al. (2019).

The OS curves for the population with a current or prior diagnosis of HF estimate that this population have a longer life expectancy than the population with or without a current or prior diagnosis of HF. It is likely that this is caused by the use of immature data to model survival curves and is a limitation of the model. As a result of this, the model predicts that the TriageHF Plus is more cost-effective when utilised in a population with a current or prior diagnosis of HF, compared to the total population eligible for TriageHF Plus. A panel of experts selected the Weibull curve as the base case.

Table S6: Overall Survival for the TriageHF Plus population

| **Survival** | **1-year survival** | **5-year survival** | **10-year survival** | **AIC** | **BIC** |
| --- | --- | --- | --- | --- | --- |
| Total population survival curve estimates | | | |  |  |
| Exponential | 96.0% | 81.4% | 66.2% | 385 | 389 |
| Weibull (base case) | 96.2% | 57.1% | 16.8% | 383 | 391 |
| Gompertz | 96.3% | 2.5% | 0.0% | 385 | 393 |
| Log-normal | 96.0% | 73.3% | 55.2% | 381 | 390 |
| Log-logistic | 96.2% | 62.5% | 34.0% | 382 | 391 |
| Generalised gamma | Did not converge | | | | |
| Population diagnosed with HF survival curve estimates | | | |  |  |
| Exponential | 95.9% | 81.2% | 65.9% | 267 | 270 |
| Weibull (scenario) | 96.3% | 59.5% | 20.1% | 229 | 236 |
| Gompertz | 96.3% | 4.4% | 0.0% | 230 | 237 |
| Log-normal | 96.0% | 74.0% | 56.5% | 229 | 237 |
| Log-logistic | 96.2% | 63.8% | 35.8% | 229 | 237 |
| Generalised gamma | Did not converge | | | | |

*AIC= Akaike information criterion, BIC= Bayesian information criterion.*

# Section 3. Health-related quality of life: utility decrement for hospitalisation by NYHA

The mean utility decrement split by NHYA which informs the utilities used to calculated health-adjusted life years of the cohort is shown in **Table S7**.

Table S7: Mean utility decrement for the different NYHA classifications along with the source of the decrements.

| **Parameter** | **Mean utility decrement** | **Source** |
| --- | --- | --- |
| No diagnosis of heart failure | -0.040 | Derived from Griffiths et al. 2014 [2]. |
| NYHA class I | -0.040 |  |
| NYHA class II | -0.070 |  |
| NYHA class III | -0.100 |  |
| NYHA class IV | -0.290 |  |
| Weighted average utility decrement | -0.070 | Derived |

NYHA*= New York Heart Association Functional Classification.*

# Section 4. Healthcare Touchpoint Episode Selection and Costings

The three healthcare touchpoints explored in this paper were all cause hospitalisations, ED visits, and OP appointments.

## Hospitalisations

**Figure S1** outlines the selection process for hospitalisations from NHS secondary care data.

**Figure S1: Admitted patient care episodes: detailed selection process**

*APC= Admitted Patient Care, ADMIMETH= Method of admission into hospital, DISDATE= Date of Discharge, SUSHRG= Secondary Uses Services Healthcare Resource Group, ADMIDATE= Date of Admission*

SUSHRG national tariff codes were used to extract cost of APC episode. SSEM tariffs are used within the NHS costing structures when a person completes their treatment faster than what is expected. These were applied when an episode fulfilled the following criteria:

1. The ADMIMETH: 21, 22, 23, 24, 25, 28, 2A, 2B, 2C, or 2D [NHS Data dictionary]
2. Patient 19 years or older at time of admission
3. The SUSHRG code applicable for SSEM [NHS Data dictionary]
4. The average stay for that SUSHRG code must be greater than 2 days
5. Episode length of stay 0 (discharge same day) or 1 (discharged following day)

For APC episodes where SUSHRG codes were not defined in the national tariff, two clinicians (JKT and FA) selected the most clinically suitable alternative diagnosis using a conservative approach to admission complexity and co-morbidity status (see **Table S7***)*. Note costing where hospitalisation resulted in death were coded as normal.

|  | **SUSHRG code^1^** | **Associated ICD-10 codes** | **Alternative** | **Rationale** |
| --- | --- | --- | --- | --- |
| 1 | DX01A,  COVID-19 Infection, with Major Manifestations, 19 years and over | U07. 1: COVID-19, confirmed by laboratory testing  J128-, Other viral pneumonia | DZ11U: Lobar, Atypical or Viral Pneumonia, without Interventions, CC Score 4-6 | Viral pneumonia ICD10 code, mid-range CC score |
| 2 | DX01A,  COVID-19 Infection, with Major Manifestations, 19 years and over | U07. 1: COVID-19, confirmed by laboratory testing  J449-, Chronic obstructive pulmonary disease, unspecified | DZ22P: Unspecified Acute Lower Respiratory Infection without Interventions, CC Score 5-8 | No pneumonia coding, assume lower respiratory tract infection |
| 3 | UZ01Z, Data Invalid for Grouping | Null (Primary diagnosis) | Exclude | Exclude |
| 4 | WD08Z, Mental and Behavioural Disorders Due to Drug or Alcohol Use | F101- , Mental and behavioural disorders due to use of alcohol Withdrawal state | WH03A: Toxic Effect of Other Substance with CC Score 2+ | Closest alcohol withdrawal state code identified |
| 5 | WD01Z, Delirium, treated by a Non-Specialist Mental Health Service Provider | I500-, Delirium due to known physiological condition | AA25F: Cerebral Degenerations or Miscellaneous Disorders of Nervous System, CC Score 5-7 | Closest delirium code identified |

Table S8: Uncosted SUSHRG codes – alternative codes

*CC = co-morbidity code, SUSHRG= Secondary Uses Services Healthcare Resource Group, APC= Admitted Patient Care, ICD10= International Classification of Diseases 10^th^ Revision*

## Emergency Department visits

### Methods

**Figure S2** outlines the selection process for emergency department (ED) visits.

Figure S2: A flowchart showing the filtering and selection process for ED visits

ED= Emergency Department, ARRIVAL_DATE= Date of arrival, ADMIMETH= Method of admission, ARRIVAL_TIME= Time of arrival, SUSHRG= Secondary Uses Services Healthcare Resource Group.

Inverse-probability-of-treatment weighting was used to balance the cohorts to estimate the impact of TriageHF on the rate of ED attendances. The variables were selected to align with previous weighting used on the same dataset, the variables included were age, gender, device type, atrial fibrillation/ flutter, IHD, ACHS, Ablation, Diabetes, CKD stage, LVEF, NYHA group and prior ED visit between March 2019 and September 2019, variables included were selected based on clinical knowledge. Where data was missing multiple predictive mean matching was used to impute 20 possible values using the same variables from the weighting using predictive mean matching. Of the 758 participants 56 had at least one missing piece of data, the proportion of missingness for each confounder ranged from 0% to 2.9%.The mean probability of enrolment was taken from these data sets by creating 20 generalised linear models and averaging the resulting probability of being in the triage group. Weights were calculated using 1/propensity score for the triage cohort, and 1/(1-propensity score) for the usual care cohort. The resulting weights had a mean of 2.023. Standardised mean differences were used to assess the balance of the weighted cohort and it was found that the weighting process improved balance with all confounders having a standardised mean difference less than 0.1 after adjustment. The inverse weights were used in a mixed effects model with negative binomial distribution to model the monthly rate of ED visits. Both the total follow up and pre-COVID19 follow up models included a two state variable representing if a device had been implanted within three months of the start of the month the ED visit takes place in. The total follow up included a variable which split the months into a pre and during COVID-19 state.

National tariffs were used to estimate the median cost of ED attendance across the two cohorts. Due to data for type of department being incomplete, the costs have been calculated using the assumption all visits took place in a type 1 or 2 department, a sensitivity analysis using a 50/50 split of department type 1 or 2 and department type 3 was conducted.

### Results

Costs are summarised in **Table S9**. Where counts were less than 5 they have been reported as such due to information governance regulations required by NHS Digital.

Table S9: Median costs of the ED attendances split by treatment group and follow up.

|  | | **Triage HF+ Cohort** | **Comparator Cohort** | **Total Cohort** |
| --- | --- | --- | --- | --- |
| Total follow-up | Total Number | 146 | 240 | 386 |
|  | Median Cost (IQR) | £165.00 (£112.00, £173.00) | £165.00 (£112.00, £173.00) | £165.00 (£112.00, £173.00) |
|  | NA | 4 | 3 | 7 |
| Total follow-up sensitivity analysis | Total Number | 146 | 240 | 386 |
|  | Median Cost (IQR) | £121.00 (£94.50, £125.00) | £121.00 (£94.50, £125.00) | £121.00 (£94.50, £125.00) |
|  | NA | 4 | 3 | 7 |
| Pre-COVID-19 follow-up Time Horizon | Total Number | 31 | 104 | 135 |
|  | Median Cost (IQR) | £165.00 (£118.50, £173.00 | £165.00 (£112.00, £173.00) | £165.00 (£112.00, £173.00) |
|  | NA | 1 | 1 | 2 |
| Pre-COVID-19 follow-up sensitivity analysis | Total Number | 31 | 104 | 135 |
|  | Median Cost (IQR) | £121.00 (£97.25, £125.00) | £121.00 (£94.50, £125.00) | £121.00 (£94.50, £125.00) |
|  | NA | 1 | 1 | 2 |

Table 9 IQR= Interquartile Range

## Outpatient Visits

### Methods

**Figure S3** outlines the selection process for outpatient (OP) clinic visits.

Figure S3: Outpatient Visits: detailed selection process

*OP=Outpatient, MAINSPEF= Department of outpatient appointment, APPTDATE= Appointment Date, STUDY_ID= Unique identifier.*

The same propensity scoring methods were applied to the data to estimate the impact of TriageHF+ on OP appointments, prior outpatient appointment between March 2019 and September 2019 replaces the prior ED attendances.

Costs of additional outpatient appointments were estimated separately when resource use in the case of a high alert was considered.

# Section 5. Economic modelling, additional results

**Tables S11 – S12** and **Figures S4-7** provide additional results of the economic modelling, as referenced to in the main text.

Table S11. Estimated mean (95% UI) incremental costs by site size

| **Parameter** | **Incremental costs** | | | |
| --- | --- | --- | --- | --- |
|  | **Per-person** | **Site size: 50** | **Site size: 100** | **Site size: 300** |
| Base-case parameters^1^ | -£3,980  (-£5,548 to -£1,844) | -£199,000  (-£212,096 to -£185,904) | -£398,000  (-£416,520 to -£379,480) | -£1,194,000  (-£1,226,078 to -£1,161,922) |
| 1-year time horizon | -£707  (-£991 to -£318) | -£35,350  (-£37,729 to -£32,971) | -£70,700  (-£74,065 to -£67,335) | -£212,100  (-£217,928 to -£206,272) |
| 5-year time horizon | -£2,760  (-£3,871 to -£1,362) | -£138,000  (-£146,871 to -£129,129) | -£276,000  (-£288,545 to -£263,455) | -£828,000  (-£849,729 to -£806,271) |
| Pre-COVID-19 | -£2,901  (-£4,971 to -£130) | -£145,050  (-£162,166 to -£127,934) | -£290,100  (-£314,305 to -£265,895) | -£870,300  (-£912,224 to -£828,376) |
| HF population | -£4,220  (-£5,858 to -£1,876) | -£211,000  (-£225,078 to -£196,922) | -£422,000  (-£441,910 to -£402,090) | -£1,266,000  (-£1,300,485 to -£1,231,515) |
| CRT-D subgroup | -£2,277  (-£3,946 to -£805) | -£113,850  (-£124,955 to -£102,745) | -£227,700  (-£243,405 to -£211,995) | -£683,100  (-£710,302 to -£655,898) |
| CRT-P subgroup | -£2,410  (-£4,361 to -£700) | -£120,500  (-£133,444 to -£107,556) | -£241,000  (-£259,305 to -£222,695) | -£723,000  (-£754,705 to -£691,295) |
| ICD subgroup | -£3,441  (-£6,412 to -£1,066) | -£172,050  (-£190,951 to -£153,149) | -£344,100  (-£370,830 to -£317,370) | -£1,032,300  (-£1,078,598 to -£986,002) |

*^1^ Total follow-up, total population, lifetime horizon. U****I=*** *uncertainty interval; HF= heart failure, CRT-D= cardiac resynchronisation therapy with a defibrillator, CRT-P Cardiac resynchronisation therapy device with a pacemaker, ICD= Implantable cardioverter defibrillator*

Table S12. Estimated mean (95% UI) NMB by site size

| **Parameter** | **Net monetary benefit** | | | |
| --- | --- | --- | --- | --- |
|  | **Per-person** | **Site size: 50** | **Site size: 100** | **Site size: 300** |
| Base-case parameters^1^ | £4,168  (£1,909 to £5,800) | £208,400  (£194,643 to £222,157) | £416,800  (£397,345 to £436,255) | £1,250,400  (£1,216,703 to £1,284,097) |
| 1-year time horizon | £740  (£337 to £1,034) | £37,000  (£34,536 to £39,464) | £74,000  (£70,515 to £77,485) | £222,000  (£215,964 to £228,036) |
| 5-year time horizon | £2,885  (£1,433 to £4,049) | £144,250  (£135,001 to £153,499) | £288,500  (£275,420 to £301,580) | £865,500  (£842,845 to £888,155) |
| Pre-COVID-19 | £3,043  (£162 to £5,199) | £152,150  (£134,342 to £169,958) | £304,300  (£279,115 to £329,485) | £912,900  (£869,278 to £956,522) |
| HF population | £4,417  (£1,982 to £6,118) | £220,850  (£206,227 to £235,473) | £441,700  (£421,020 to £462,380) | £1,325,100  (£1,289,281 to £1,360,919) |
| CRT-D subgroup | £2,389  (£863 to £4,119) | £119,450  (£107,938 to £130,962) | £238,900  (£222,620 to £255,180) | £716,700  (£688,502 to £744,898) |
| CRT-P subgroup | £2,530  (£754 to £4,562) | £126,500  (£113,037 to £139,963) | £253,000  (£233,960 to £272,040) | £759,000  (£726,022 to £791,978) |
| ICD subgroup | £3,595  (£1,139 to £6,685) | £179,750  (£160,142 to £199,358) | £359,500  (£331,770 to £387,230) | £1,078,500  (£1,030,470 to £1,126,530) |

*^1^ Total follow-up, total population, lifetime horizon. UI= uncertainty interval; HF= heart failure; NMB= net monetary benefit, CRT-D= cardiac resynchronisation therapy with a defibrillator, CRT-P Cardiac resynchronisation therapy device with a pacemaker, ICD= Implantable cardioverter defibrillator*

**Figure S4. Cost-neutrality IRR breakeven analysis in scenarios for the base case, pre-COVID-19 follow-up and the HF population**

*IRR=Incidence Ratio Rate, HF= Heart Failure*

**Figure S5. Cost-neutrality IRR breakeven analysis in scenarios by CIED type**

*CRT-D= cardiac resynchronisation therapy with a defibrillator, CRT-P Cardiac resynchronisation therapy device with a pacemaker, ICD= Implantable cardioverter defibrillator, IRR= Incidence Ratio Rate*

**Figure S6. Cost-effectiveness IRR breakeven analysis in scenarios for the base case, pre-COVID-19 follow-up and the HF population**

*IRR=Incidence Ratio Rate, HF= Heart Failure*

**Figure S7. Cost- effectiveness IRR breakeven analysis in scenarios by CIED type**

*CRT-D= cardiac resynchronisation therapy with a defibrillator, CRT-P Cardiac resynchronisation therapy device with a pacemaker, ICD= Implantable cardioverter defibrillator, IRR= Incidence Ratio Rate*

# Section 6. Resources Use and Costings

Throughout the study those in the TriageHF+ pathway triggered HF alerts which resulted in additional resource use the table below outlines the resource use in terms of the HF alerts received. Time required for staff to perform the consultations are reported in minutes in the format mean (standard deviation). Where none of the allotted staff members of a given band monitoring high alerts performed consultation it was assumed that no time was spent by that band calling the participant.

## TriageHF Plus cohort

**Tables S13** and **S14** provide additional detail for costing used in the economic model for the TriageHF Plus cohort.

Table S13: Resource use for HF alerts

| **Covariable** | **Total follow-up (base case) n=406** | **Pre-COVID-19 follow-up n=99** | **HF population n=391** |
| --- | --- | --- | --- |
| **Low / medium risk and standard care: Market share across pharmacological treatments** | | | |
| Beta Blocker (Carvedilol) | 85.4% | 84.6% | 87.6% |
| ACE-i/ARB/ARNI (Ramipril) | 73.2% | 74.1% | 76.8% |
| Diuretic (Furosemide) | 49.3% | 50.1% | 52.3% |
| MRA (Spironolactone) | 44.1% | 43.7% | 47.8% |
| **High-risk: Market share across pharmacological treatments** | | | |
| Beta Blocker (Carvedilol) | 92.2% | 95.3% | 91.9% |
| ACE-i/ARB/ARNI (Ramipril) | 78.1% | 76.7% | 79.0% |
| Diuretic (Furosemide) | 64.1% | 60.5% | 66.1% |
| MRA (Spironolactone) | 56.3% | 58.1% | 58.1% |
| **High-risk: Initial consultation** | | | |
| Initial consultation (%) | 52.2% (212/406) | 56.6% (56/99) | 52.4% (205/391) |
| Nurse band 4 time required | 13.17 (0.86) | 10.33 (1.08) | 13.17 (0.86) |
| Nurse band 5 time required | 0.00 (0.00) | 0.00 (0.00) | 0.00 (0.00) |
| Nurse band 6 time required | 7.85 (0.55) | 9.87 (1.05) | 7.90 (0.60) |
| Nurse band 7 time required | 8.40 (0.76) | 9.33 (2.50) | 8.35 (0.77) |
| Nurse band 8a time required | 18.97 (1.85) | 14.13 (2.55) | 18.97 (1.85) |
| Nurse band 8ab time required | 0.00 (0.00) | 0.00 (0.00) | 0.00 (0.00) |
| Nurse band 8c time required | 0.00 (0.00) | 0.00 (0.00) | 0.00 (0.00) |
| Nurse band 8d time required | 0.00 (0.00) | 0.00 (0.00) | 0.00 (0.00) |
| Nurse band 9 time required | 0.00 (0.00) | 0.00 (0.00) | 0.00 (0.00) |
| Consultant time required | 11.20 (1.14) | 14.67 (5.94) | 11.20 (1.14) |
| **High-risk: Follow-up consultation** | | | |
| Follow-up consultations (%) | 43.8% (178/406) | 44.4% (44/99) | 44.2% (173/391) |
| Nurse band 4 time required | 10.75 (0.82) | 8.65 (1.51) | 10.75 (0.82) |
| Nurse band 5 time required | 0.00 (0.00) | 0.00 (0.00) | 0.00 (0.00) |
| Nurse band 6 time required | 6.68 (0.50) | 9.17 (1.50) | 6.80 (0.52) |
| Nurse band 7 time required | 7.43 (0.75) | 5.00 (0.45) | 7.50 (0.79) |
| Nurse band 8a time required | 16.40 (1.28) | 8.60 (1.33) | 16.40 (1.28) |
| Nurse band 8ab time required | 0.00 (0.00) | 0.00 (0.00) | 0.00 (0.00) |
| Nurse band 8c time required | 0.00 (0.00) | 0.00 (0.00) | 0.00 (0.00) |
| Nurse band 8d time required | 0.00 (0.00) | 0.00 (0.00) | 0.00 (0.00) |
| Nurse band 9 time required | 0.00 (0.00) | 0.00 (0.00) | 0.00 (0.00) |
| Consultant time required | 9.73 (1.29) | 10.0 (1.00) | 0.40 (1.21) |

*ACE-i= Angiotensin-converting enzyme inhibitors, ARB= angiotensin II receptor blockers, ARNI= angiotensin receptor- neprilysin inhibitor , MRA= mineralocorticoid receptor antagonist, HF= Heart Failure*

Table S14: Costs associated with resources used in the TriageHF Plus pathway with source of cost.

| **Parameter** | **Cost** | **Source** |
| --- | --- | --- |
| **Staff costs** | | |
| Band 4 nurse | £42 per hour | Jones et al. 2023 [3]. Hourly costs for qualified nurses including qualifications. |
| Band 5 nurse | £52 per hour |  |
| Band 6 nurse | £63 per hour |  |
| Band 7 nurse | £74 per hour |  |
| Band 8a nurse | £83 per hour |  |
| Band 8b nurse | £96 per hour |  |
| Band 8c nurse | £113 per hour |  |
| Band 8d nurse | £133 per hour |  |
| Band 9 nurse | £157 per hour |  |
| Consultant | £157 per hour |  |
| **Clinical investigations** | | |
| Plasma creatine/ blood urea nitrogen | £4.70 per test | NHS National Cost Collection 2021/22 [4]. Code: DAPS08. Assumed to be equal for all blood tests. |
| Blood count tests | £4.70 per test |  |
| Cardiac enzymes | £4.70 per test |  |
| ECG | £70 per test | NHS National Cost Collection 2021/22 [4]. Code: EY50Z. DADS. |
| Chest x-ray | £38 per test | NHS National Cost Collection 2021/22 [4]. Code: DAPF. DADS. |
| Echocardiography | £70 per test | NHS National Cost Collection 2021/22 [4]. Code: EY50Z. DADS. |
| **Medication costs*** | | |
| ACE-I (Ramipril, 10 mg) | £0.39 per 28-tablet pack | Gov.UK. e-MIT [5]. |
| Beta blocker (Carvedilol, 50 mg) | £0.63 per 28-tablet pack |  |
| MRA (Spironolactone, 50 mg) | £1.67 per 28-tablet pack |  |
| Diuretic (Furosemide, 40 mg) | £0.45 per 28-tablet pack |  |
| **Healthcare utilisation** | | |
| All-cause non-elective hospitalisation episode | £2,762 per episode | Median cost of hospitalisation derived in study [6]. |
| HF non-elective long stay (NEL) inpatient hospitalisation costs | £3,758 per episode | NHS National Cost Collection 2021/22 [4]. National average (NEL). |
| HF non-elective short stay (NES) inpatient hospitalisation costs | £666 per episode | NHS National Cost Collection 2021/22 [4]. National average (NES). |
| Cardiologist outpatient appointment | £200 per appointment | NHS National Cost Collection 2021/22 [4]. Code: WF01B. Cardiology service (OP). |

*CPRD=Clinical Practice Research Datalink, ECG= electrocardiogram, ED= Emergency department, OP= Outpatient, EL= elective inpatient GP= general practitioner, HF= heart failure, NEL= non-elective long stay, NES= non-elective short stay, NHS= National Health Service, ACE-i= Angiotensin-converting enzyme inhibitors, MRA= mineralocorticoid receptor antagonist, EMS= Electric Monitoring Service, e-MIT=electronic market information tool.*

## Standard care

Resource costs associated with usual care (including “low” and “medium” risk remote monitoring patients in the TriageHF Plus treatment group). were inferred from national guidelines. Assumptions for usual care are detailed in **Table S15**.

Table S15 Monthly monitoring costs for the usual care cohort presented as mean (SE)

| **Parameter** | **Mean (SE)** | **Source and method** |
| --- | --- | --- |
| **Frequency of visits per month** | | |
| GP visit | 1.928 (0.193) | Mean annual use is derived using UK-specific data (Clinical Practice Research Datalink) and converted to monthly frequencies [7]. |
| Cardiologist visit | 0.004 (0.000) |  |
| Other physician visit | 0.030 (0.003) |  |
| ED referral | 0.001 (0.000) |  |
| **Frequencies of clinical tests per month** | | |
| Plasma urea /creatine | 0.333 (0.033) | Published recommendations for the frequency of routine clinical investigations based on European Society of Cardiology 2005 guidelines [8]. The frequency per person per year is informed by Abraham et al. 2011 [9]. |
| Blood count tests | 0.083 (0.008) |  |
| ECG | 0.027 (0.003) |  |
| Chest x-ray | 0.027 (0.003) |  |
| Echocardiography | 0.027 (0.003) |  |
| Cardiac enzymes | 0.027 (0.003) |  |

*GP= general practitioner, SE= standard error, ECD-Electrocardiogram.*

# Section 7: Acknowledgements

The TriageHF Plus team:

*Northern Care Alliance*: Nicola Conway, Wendy Phillips, Timothy Latchford, Daniel Terry, Stephen Williams. Lisa Docherty, Bridget Galaska, Abigale Irvine, Natalie Spanswick, Sama Nandakumar, Catherine McClelland, Sabah Riaz, Marie Pye, Sarah Constable, Nicola Lee, Janet Mills-Cook, Diane Livesay, Mary Kathleen Fitzgerald, Ranjin Thomas, Jane Amaeshi, Sarah Whittaker, David Strong, Janette Hoy, Pamela Bradley, Julie Melville, Linda Johnson, Mohammed Mahmood, Carol Lunney, Claire Carty, Matthew Kahn Mita Kale and Yuran Zheng.

*Wythenshawe Hospital*: Paul Callan, Sue Ewings, Hannah Garnett, Carol Crosbie, Kathryn Hulley

*Manchester Royal Infirmary:* Christina Muldoon, Hayley Armitage, Laura Wockwar, Sophie Rooker, Claire Ashworth, Kelly Hogan, Ana Domingos, Amy Brett, Michelle Page, Manju Jose, Colin Cunnington

# Supplementary Material references

1. *Briggs AKC, Sculpher M. Decision modelling for health economic evaluation: Oxford: Oxford University Press; 2006.*
2. Griffiths, A., et al., *The cost effectiveness of ivabradine in the treatment of chronic heart failure from the UK National Health Service perspective.* Heart, 2014. **100**(13): p. 1031-1036.
3. *Jones KC, Weatherly H, Birch S, Castelli A, Chalkley M, Dargan A, et al. Unit Costs of Health and Social Care 2022 Manual. 2022.*
4. *National Health Service (NHS) England. National Cost Collection 2021/22. 2023 Available from:* [*https://www.england.nhs.uk/publication/2021-22-national-cost-collection-data-publication/*](https://www.england.nhs.uk/publication/2021-22-national-cost-collection-data-publication/)*.*
5. *Department of Health and Social Care. eMIT national database. 2011 Available from: https://www.gov.uk/government/publications/drugs-and-pharmaceutical-electronic-market-information-emit*
6. Ahmed, F.Z., et al., *Association of a device‐based remote management heart failure pathway with outcomes: TriageHF Plus real‐world evaluation.* ESC Heart Failure, 2024. **11**(5): p. 2637-2647.
7. McMurray, J.J., et al., *Cost-effectiveness of sacubitril/valsartan in the treatment of heart failure with reduced ejection fraction.* Heart, 2018. **104**(12): p. 1006-1013.
8. Nicholls, M.G. and A.M. Richards, *Disease monitoring of patients with chronic heart failure.* Heart, 2007. **93**(4): p. 519-523.
9. Abraham, W.T., et al., *Intrathoracic impedance vs daily weight monitoring for predicting worsening heart failure events: results of the Fluid Accumulation Status Trial (FAST).* Congestive Heart Failure, 2011. **17**(2): p. 51-55.
